# Fruit and Soil Quality of Organic and Conventional Strawberry Agroecosystems

**DOI:** 10.1371/journal.pone.0012346

**Published:** 2010-09-01

**Authors:** John P. Reganold, Preston K. Andrews, Jennifer R. Reeve, Lynne Carpenter-Boggs, Christopher W. Schadt, J. Richard Alldredge, Carolyn F. Ross, Neal M. Davies, Jizhong Zhou

**Affiliations:** 1 Department of Crop and Soil Sciences, Washington State University, Pullman, Washington, United States of America; 2 Department of Horticulture and Landscape Architecture, Washington State University, Pullman, Washington, United States of America; 3 Department of Plants, Soils and Climate, Utah State University, Logan, Utah, United States of America; 4 Center for Sustaining Agriculture and Natural Resources, Washington State University, Pullman, Washington, United States of America; 5 Biosciences Division, Oak Ridge National Laboratory, Oak Ridge, Tennessee, United States of America; 6 Department of Statistics, Washington State University, Pullman, Washington, United States of America; 7 School of Food Science, Washington State University, Pullman, Washington, United States of America; 8 Department of Pharmaceutical Sciences, Washington State University, Pullman, Washington, United States of America; 9 Department of Botany and Microbiology, Institute for Environmental Genomics, University of Oklahoma, Norman, Oklahoma, United States of America; Cairo University, Egypt

## Abstract

**Background:**

Sale of organic foods is one of the fastest growing market segments within the global food industry. People often buy organic food because they believe organic farms produce more nutritious and better tasting food from healthier soils. Here we tested if there are significant differences in fruit and soil quality from 13 pairs of commercial organic and conventional strawberry agroecosystems in California.

**Methodology/Principal Findings:**

At multiple sampling times for two years, we evaluated three varieties of strawberries for mineral elements, shelf life, phytochemical composition, and organoleptic properties. We also analyzed traditional soil properties and soil DNA using microarray technology. We found that the organic farms had strawberries with longer shelf life, greater dry matter, and higher antioxidant activity and concentrations of ascorbic acid and phenolic compounds, but lower concentrations of phosphorus and potassium. In one variety, sensory panels judged organic strawberries to be sweeter and have better flavor, overall acceptance, and appearance than their conventional counterparts. We also found the organically farmed soils to have more total carbon and nitrogen, greater microbial biomass and activity, and higher concentrations of micronutrients. Organically farmed soils also exhibited greater numbers of endemic genes and greater functional gene abundance and diversity for several biogeochemical processes, such as nitrogen fixation and pesticide degradation.

**Conclusions/Significance:**

Our findings show that the organic strawberry farms produced higher quality fruit and that their higher quality soils may have greater microbial functional capability and resilience to stress. These findings justify additional investigations aimed at detecting and quantifying such effects and their interactions.

## Introduction

Although global demand for organic products remains robust, consumer demand for these products is concentrated in North America and Europe [1]. For example, in the United States, which ranks fourth in organically farmed land globally [1], organic food sales have increased by almost a factor of six, from $3.6 billion in 1997 to $21.1 billion in 2008 (or more than 3 percent of total U.S. food sales) [2]. More than two-thirds of U.S. consumers buy organic products at least occasionally, and 28 percent buy organic products weekly [2]. Three of the most important reasons consumers purchase organic foods are health benefits (i.e., less pesticide residues and greater nutrition), taste, and environmentally friendly farming practices, such as those that promote soil health [3].

While there is strong evidence that organic foods have significantly less pesticide residues [4]–[6], this is not the case for organic foods being more nutritious. Although there is no universally accepted definition of what constitutes a nutritious food, recent scientific opinion has stressed that more nutritious foods are those that are more nutrient dense relative to their energy contents [7]. Although carbohydrates and fats are considered essential nutrients, the current concept of nutrient dense foods, and hence more nutritious foods, places the emphasis on foods that contain more protein, fiber, vitamins, or minerals, as well as specific phytochemicals, such as the polyphenolic antioxidants found in fruits and vegetables [8].

In the past 10 years, ten review studies of the scientific literature comparing the nutrition of organic and conventional foods have been published. Eight of these review studies [9], [10], [11], [12], [13], [14], [15], [16] found some evidence of organic food being more nutritious, whereas two review articles [17], [18] concluded that there were no consistent nutritional differences between organic and conventional foods. Comparisons of foods from organic and conventional systems are often complicated by the interactive effects of farming practices, soil quality, plant varieties, and the time of harvest on nutritional quality. Hence, many of the comparative studies cited in some of the earlier reviews were not experimentally well designed to draw valid conclusions [13], [17]; for example, soils or crop varieties were not the same on each organic/conventional field pair. The few studies that have compared organic and conventional foods for their organoleptic (sensory) properties have shown mixed results or used unreliable experimental designs [14], [17].

A widely accepted definition of soil quality is the capacity of a soil to sustain biological productivity, maintain environmental quality, and promote plant and animal health [19]. Soil quality may be inferred from measurable soil properties termed soil quality indicators [20]. Organic farming practices compared to conventional farming practices have been shown to improve soil quality indicators based on traditional measures of biological, chemical, and physical properties [21], [22], [23], with few studies showing no advantages [24]. However, traditional measures inadequately assess the roles of microbial community structure and genetic diversity in soil ecosystem processes that directly impact soil quality [25]. Examples of important soil ecosystem processes facilitated by microorganisms include nitrogen fixation, denitrification, pesticide degradation, and other organic xenobiotic degradation. Soil DNA analysis using microarray technology can target those microbial genes involved in specific soil ecosystem processes and measure their abundance and diversity [26], [27], allowing a more complete investigation of soil quality.

The majority of previous organic/conventional studies have focused on either comparing fruit quality or soil quality. The few studies that have compared both facets have limited their analyses to selected properties. Currently, no published study has integrated interdisciplinary knowledge and robust methodologies in a systems approach to quantitatively compare a comprehensive range of both fruit and soil quality indices using multiple organic and conventional farms, multiple varieties, and multiple sampling times. Here, we assembled an interdisciplinary team of scientists representing agroecology, soil science, microbial ecology, genetics, pomology, food chemistry, sensory science, and statistics to address the following question: Are there significant differences in nutritional and organoleptic fruit properties and in soil quality, including soil ecosystem functional genes, between commercial organic and conventional strawberry agroecosystems?

Although some farm production conditions can be simulated at research stations, farming systems research that measures multiple variables can often only be properly studied under actual farming or agroecosystem conditions [28]. Thus, our study's experimental units are real commercial organic and conventional strawberry farms, located in California. We chose to study strawberries (*Fragaria x ananassa* Duch.) as the food of choice because of their high economic value as a fruit crop, documented nutritional benefits, popularity in the consumer diet, and suitability for sensory evaluation. California is an appropriate location for the commercial strawberry farms in our study because it is the leading producer, accounting for more than 25% of the world's strawberry production [29], [30], with nearly 5% of its total strawberry acreage in organic production [29].

To determine if differences in food and soil quality exist, we sampled repeatedly harvested strawberry varieties (‘Diamante’, ‘San Juan’, and ‘Lanai’) and soils at multiple sampling times in 2004 and 2005 from 13 pairs of adjacent organic and conventional fields from commercial farms. Each organic/conventional field pair had the same soil type and the same strawberry variety planted at similar times. Because strawberries go through different growth cycles during the 7-month harvest season, we analyzed 42 fruit, 11 leaf, and 6 organoleptic properties multiple times during the two years of our study. Strawberries in each field pair were analyzed at the same time and stage of harvest maturity, and under identical storage conditions and transportation methods so that the strawberries were as close to retail consumption as possible. In addition to measuring 31 traditional soil chemical and biological properties, we analyzed soil DNA using microarray technology to target those microbial genes involved in 11 specific ecosystem processes.

## Results and Discussion

### Strawberry Quality

Strawberry leaves were analyzed for plant nutrients and fruit were analyzed for plant nutrients, fruit quality, nutritional value, and organoleptic properties. Leaf P and fruit P and K concentrations were significantly higher in conventionally grown strawberry plants than in organically grown plants (Table 1); leaf Mg and fruit N were also notably higher (*P*<0.10) in conventionally grown strawberry plants. All other strawberry and leaf nutrient concentrations were similar. While there are no published recommendations for optimum levels of foliar concentrations of mineral nutrients for strawberries grown in California, all farm fields were fertilized according to local industry standards, as recommended by professional horticulturists. No nutrient deficiency or toxicity symptoms were observed on organically or conventionally grown strawberry plants during the two growing seasons.

**Table 1:** Mineral elements (mean ± standard error) in strawberry leaves and fruit from organic (ORG) and conventional (CON) farms (n = 13).

Mineral Element	‘Diamante’		‘Lanai’		‘San Juan’		*P* Value
	ORG	CON	ORG	CON	ORG	CON	
	Leaves						
Nitrogen (% FW)	2.51±0.10	2.76*±0.10	3.06±0.12	2.91±0.12	2.84±0.11	2.84±0.11	0.020
Calcium (% FW)	0.73±0.36	1.19†±0.36	0.81±0.37	0.88±0.37	0.87±0.37	0.77±0.37	0.036
	ORG			CON			
Phosphorus‡ (% FW)	0.37±0.016			0.45†±0.016			0.001
Potassium‡ (% FW)	1.56±0.04			1.58±0.04			0.71
Sulfur (% FW)	0.215±0.009			0.214±0.009			0.91
Magnesium (% FW)	0.311±0.047			0.354*±0.047			0.066
Boron (ppm)	38.9±2.36			38.7±2.36			0.95
Zinc (ppm)	59.9±1.31			63.3±1.31			0.73
Manganese (ppm)	128±34.5			182±34.5			0.19
Copper (ppm)	5.24±1.46			4.81±1.46			0.14
Iron (ppm)	207±24.1			214±24.1			0.70
	Fruit						
Nitrogen (% FW)	1.02±0.11			1.08*±0.11			0.078
Phosphorus (% FW)	0.247±0.012			0.286†±0.012			0.001
Potassium (% FW)	1.50±0.05			1.65^**^±0.05			0.010
Calcium (% FW)	0.120±0.015			0.132±0.015			0.18
Magnesium (% FW)	0.130±0.003			0.134±0.003			0.26
Boron (ppm)	14.5±17.5			15.2±17.5			0.57
Zinc (ppm)	9.95±0.71			9.96±0.71			0.99

Leaves and fruit were sampled in June 2004 and April and June 2005 from 13 pairs of organic (ORG) and conventional (CON) farm fields. Probabilities (*P* values) for treatment x variety interactions for leaf N and Ca, and for treatment main effects for the remaining leaf and fruit mineral elements are given. Means and standard errors of mineral elements in leaves and fruit for individual sampling/harvest times, varieties, and years are listed in Tables S4A, C, and D.

*Means are notably different at *P*<0.10.

† Means are significantly different at *P*<0.01.

‡ Based on Dietary Reference Intakes (DRI) [75], a standard serving (140 g) [76] of the fresh organic strawberries would supply 8 and 4% less, respectively, of the daily phosphorus and potassium requirements of adult men and women than would the conventional strawberries.

**Figure 1:**
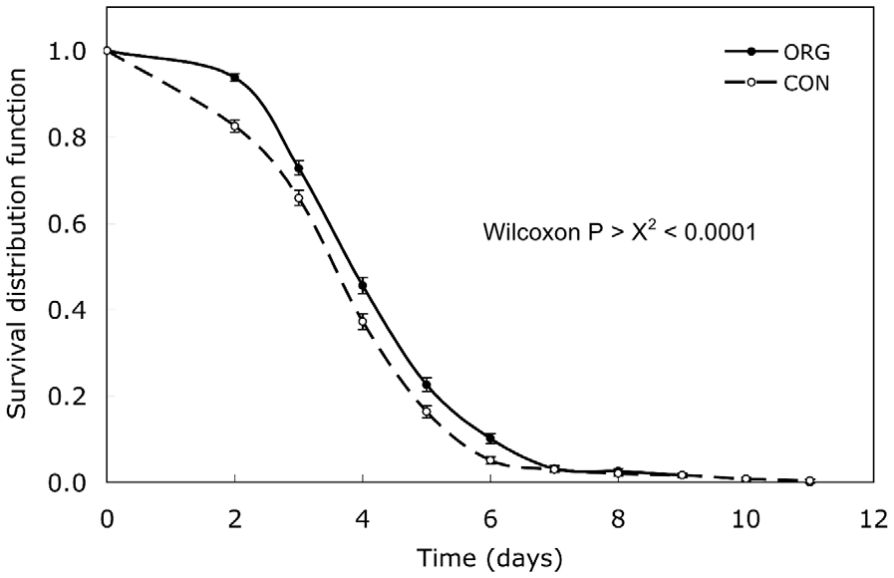
Survival distribution curves of rot tests for ‘Diamante’ and ‘San Juan’ strawberry fruit sampled from the 5 pairs of organic (ORG) and conventional (CON) farm fields in June and September 2004. Mean survival days were CON = 4.15±0.06 and ORG = 4.54±0.06. (Error bars indicate standard error.)

When susceptibility to fungal post-harvest rots was evaluated, organic strawberries had significantly longer survival times (less gray mold incidence) than conventional strawberries (Figure 1). When strawberries were exposed to a two-day shelf-life interval, the percent loss in fresh weight was significantly less for the organic berries than for the conventional berries (Table 2). These results indicate that the organic strawberries would have a longer shelf life than the conventional strawberries because of slower rotting and dehydration, perhaps due to augmentation of cuticle and epidermal cell walls. There were no fungicides applied to the organic strawberry fields for post-harvest control of gray mold (*Botrytis cinerea*), in contrast to multiple fungicide applications to the conventional fields. Although sulfur was applied to the organic fields to control powdery mildew (*Sphaerotheca macularis*), sulfur sprays are ineffective against gray mold [31]. This suggests that the organic strawberries may have been more resistant or avoided infection by means other than fungicides (e.g., systemic-acquired resistance).

**Table 2:** Fruit characteristics (mean ± standard error) of strawberries from organic and conventional farms (n = 13).

Fruit Quality Variable (units)	Organic	Conventional	*P* Value
Fruit fresh weight (g)	24.07±0.68	27.78±0.68	0.001
Dry matter (%)	10.03±0.20	9.26±0.20	0.006
Fruit weight loss (%)	25.40±5.16	27.52±5.16	0.048
Fruit firmness (N)	4.36±1.90	4.17±1.90	0.30
External L* (+60 to −60)	37.66±0.76	38.65±0.76	0.030
External C* (+60 to −60)	42.21±0.37	41.76±0.37	0.25
External h_ab_ (°)	31.26±0.63	32.14±0.63	0.048
Total antioxidant activity (mmol Trolox equivalents/g FW)	11.88±0.35	10.95±0.35	0.019
Total phenolics (mg gallic acid equivalents/g FW)	1.37±0.13	1.24±0.13	0.0003
Total ascorbic acid* (mg/g FW)	0.621±0.015	0.566±0.015	0.009
Total anthocyanins (µg P-3-Glc† equivalents/g FW)	205±19.4	192±19.4	0.103

Strawberries (‘Diamante’, ‘Lanai’, and ‘San Juan’) were sampled from 13 pairs of organic and conventional farm fields in June and September 2004 and April, June, and September 2005. Means and standard errors of fruit characteristics for individual sampling/harvest times, varieties, and years are listed in Tables S4A–E.

*Based on Dietary Reference Intakes (DRI) [75], a standard serving (140 g) [76] of the fresh organic strawberries would supply 9–10% more of the daily vitamin C (ascorbic acid) requirement of adult men and women than would the conventional strawberries.

† Pelargonidyn-3-glucoside.

Strawberries from organic farms were significantly smaller (by 13.4%) than those from conventional farms, but had significantly greater dry matter content (by 8.3%) (Table 2). Fruit firmness and external color intensity (C*) were similar between conventional and organic berries, but organic berries were darker red (significantly lower L* and h_ab_) than conventional berries. Although their darker red color did not result in a preference for the appearance of organic over conventional ‘Lanai’ and ‘San Juan’ strawberries by consumer-sensory panels, these panels did prefer the appearance of organic ‘Diamante’ berries to their conventional counterparts (Table 3).

**Table 3:** Consumer sensory evaluations (mean±standard error) of strawberries on a nine-point hedonic/intensity scale from organic (ORG) and conventional (CON) farms (n = 13).

Sensory Property	‘Diamante’		‘Lanai’		‘San Juan’		*P* Value
	ORG	CON	ORG	CON	ORG	CON	
	Hedonic/intensity ratings						
Overall acceptance	6.09 a±0.23	5.35 b±0.23	6.24 a±0.29	6.24 a±0.29	6.09 a±0.27	6.36 a±0.27	0.029
Flavor	5.95 a±0.16	5.17 b±0.16	6.08 a±0.17	5.92 a±0.17	5.86 a±0.19	6.07 a±0.19	0.044
Sweetness	5.56a±0.22	4.73 b±0.22	5.69 a±0.24	5.56 a±0.24	5.52 a±0.25	5.74 a±0.25	0.029
Appearance	6.73 a±0.37	5.97 b±0.37	6.78 a±0.39	6.97 a±0.39	7.09 a±0.39	7.03 a±0.39	0.067
	ORG			CON			
Juiciness	6.21±0.09			6.35±0.09			0.11
Tartness	4.61±0.27			4.75±0.27			0.38

Strawberry fruit (‘Diamante’, ‘Lanai’, and ‘San Juan’) were sampled from 13 pairs of organic and conventional farm fields in September 2004 and April, June, and September 2005. Differences between values within rows followed by different letters are significant at *P*<0.05. Means and standard errors of consumer sensory evaluations for individual sampling/harvest times, varieties, and years are listed in Tables S4B-E.

Organic strawberries had significantly higher total antioxidant activity (8.5% more), ascorbic acid (9.7% more), and total phenolics (10.5% more) than conventional berries (Table 2), but significantly less phosphorus (13.6% less) and potassium (9.1% less) (Table 1). Specific polyphenols, such as quercetin and ellagic acid, showed mixed or no differences (Table 4). Strawberries are among the most concentrated sources of vitamin C and other antioxidant compounds in the human diet [32]. Dietary antioxidants, including ascorbic acid (i.e., vitamin C) and phenolic compounds offer significant potential human health benefits for protection against diseases [33], [34]. For example, Olsson et al. [35] reported decreased proliferation of breast and colon cancer cells by extracts of organically grown strawberries compared to conventional berries, with ascorbic acid concentrations correlated negatively with cancer cell proliferation. Although the greater potassium concentration in the conventional strawberries is a plus, strawberries are not among the richest sources of potassium or even phosphorus [36]. Interestingly, less phosphorus in the diet may be considered desirable, given the negative effects of the increasing U.S. consumption of phosphorus [37] on vitamin D and calcium metabolism [38], and the resulting potential risk to bone health.

**Table 4:** Concentration of specific polyphenols (mean ± standard error) in strawberry fruit from organic (ORG) and conventional (CON) farms (n = 13).

Polyphenol (mg 100 g^−1^ FW)	‘Diamante’		‘Lanai’		‘San Juan’		*P* Value
	ORG	CON	ORG	CON	ORG	CON	
	April						
Quercetin glycoside	4.00±1.38	6.72*±1.38	9.18†±1.41	5.43±1.41	9.01±1.56	7.60±1.56	0.009
Quercetin, total	7.02±1.17	9.45*±1.17	11.71†±1.17	7.92±1.17	11.22±1.56	10.11±1.56	0.020
Kaempferol	0.93±0.08	1.13†±0.08	0.99±0.08	1.05±0.08	1.28*±0.10	1.07±0.10	0.026
	June						
Quercetin glycoside	6.27±1.17	7.20±1.17	2.87±1.41	6.09*±1.41	5.01±1.28	5.32±1.28	0.009
Quercetin, total	8.78±1.14	9.72±1.14	6.42±1.17	8.80*±1.17	7.92±1.15	7.81±1.15	0.020
Kaempferol	1.21‡±0.07	0.96±0.07	0.98±0.08	1.03±0.08	1.06±0.07	0.98±0.07	0.026
	September						
Quercetin glycoside	4.97±1.17	4.87±1.17	3.89±1.41	3.93±1.41	4.90±1.28	7.13*±1.28	0.009
Quercetin, total	7.51±1.14	7.33±1.14	6.61±1.17	6.57±1.17	7.37±1.15	9.19±1.15	0.020
Kaempferol	0.96±0.07	0.92±0.07	1.03±0.08	1.05±0.08	0.93±0.07	1.00±0.07	0.026
	ORG			CON			
Quercetin	2.79±0.06			2.71±0.06			0.17
Kaempferol glycoside	4.28±0.97			4.34±0.97			0.88
Kaempferol, total	5.32±1.02			5.35±1.02			0.93
Ellagic acid glycoside	55.0±13.1			53.8±13.1			0.92
Ellagic acid	2.27±1.48			2.08±1.48			0.70
Ellagic acid, total	57.2±1.31			55.9±1.31			0.88
Phloridzin glycoside	2.04±0.29			2.24±0.29			0.49
Phloretin	2.40±0.04			2.43±0.04			0.56
Phloretin, total	4.42±0.31			4.64±0.31			0.41
R-Naringin glycoside	2.90±0.95			1.35±0.95			0.27
S-Naringin glycoside	2.90±0.98			1.46±0.98			0.32
R-Naringenin	0.43±0.07			0.44±0.07			0.83
S-Naringenin	0.24±0.07			0.29±0.07			0.51
R-Naringenin, total	3.31±0.95			1.77±0.95			0.28
S-Naringenin, total	3.12±0.98			1.73±0.98			0.34

Fruit were sampled in June and September 2004 and April, June, and September 2005 from 13 pairs of organic (ORG) and conventional (CON) farm fields. Least square means ± standard error of the means. Probabilities (*P* values) for treatment x variety x month interactions for quercetin glycoside, total quercetin, and kaempferol, and for treatment main effects for the remaining polyphenols are given. Means and standard errors of specific polyphenol concentrations for individual sampling/harvest times, varieties, and years are listed in Tables S4A–E.

*Means are notably different at *P*<0.10.

† Means are significantly different at *P*<0.05.

‡ Means are significantly different at *P*<0.01.

Using hedonic/intensity ratings, consumer-sensory panels found organic ‘Diamante’ strawberries to be sweeter and have preferable flavor, appearance, and overall acceptance compared to conventional ‘Diamante’ berries (Table 3). Organic and conventional ‘Lanai’ and ‘San Juan’ berries were rated similarly. Sensory results of sweeter tasting ‘Diamante’ strawberries were confirmed by higher soluble solids content measured in the laboratory (Table 5).

**Table 5:** Soluble solids, titratable acidity (TA), soluble solids/TA ratio, reducing sugars, total sugars, and pH (mean ± standard error) of strawberry fruit from organic (ORG) and conventional (CON) farms (n = 13).

Variable	‘Diamante’		‘Lanai’		‘San Juan’		*P* Value
	ORG	CON	ORG	CON	ORG	CON	
Soluble solids (°brix)	8.97 a±0.48	7.68 b±0.48	8.98 a±0.58	9.52 a±0.58	8.96 a±0.53	8.71 a±0.53	0.091
TA (mg citric acid g^−1^ FW)	9.16 a±0.20	7.52 bc±0.20	7.18 bc±0.26	7.51 bc±0.26	6.97 c±0.24	7.79 b±0.24	0.0005
	ORG			CON			
Soluble solids/TA	1.16±0.07			1.14±0.07			0.62
Reducing sugars (mg Glc g^−1^ FW)	69.1±2.05			69.4±2.05			0.93
Total sugars (mg Glc g^−1^ FW)	73.0±2.38			78.4±2.38			0.13
pH	3.77±0.06			3.81±0.06			0.105

Fruit were sampled from 13 pairs of organic and conventional farms in June and September 2004 and April, June, and September 2005. Probabilities (*P* values) for treatment x variety interactions for soluble solids and TA and for treatment main effects for the remaining variables are given. Differences among treatments within each row followed by different letters are significant at *P*<0.05. Means and standard errors of fruit characteristics for individual sampling/harvest times, varieties, and years are listed in Tables S4A–E.

### Soil Quality

Soils were sampled and analyzed from the top (0–10 cm) and bottom (20–30 cm) of the raised mounds in June 2004 and 2005. The organically managed surface soils compared to their conventional counterparts contained significantly greater total carbon (21.6% more) and nitrogen (30.2% more) (Table 6). Organic matter (total carbon) can have a beneficial impact on soil quality, enhancing soil structure and fertility and increasing water infiltration and storage [39]. Levels of extractable nutrients were similar in the two systems, with the exception of zinc, boron, and sodium being significantly higher and iron being notably higher in the organically farmed surface soils.

**Table 6:** Soil properties (mean ± standard error) at two depths (0–10 cm and 20–30 cm) from organic and conventional strawberry farms (n = 13).

Soil Property	Organic (0–10 cm)	Conventional (0–10 cm)	*P* Value	Organic (20–30 cm)	Conventional (20–30 cm)	*P* Value
Sand (g 100 g^−1^ soil)	60.3±7.9	60.5±8.2	0.931	61.0±8.0	59.8±8.7	0.644
Silt (g 100 g^−1^ soil)	26.8±4.8	26.4±5.5	0.619	25.8±4.9	27.3±5.7	0.821
Clay (g 100 g^−1^ soil)	13.0±3.4	13.1±2.9	0.925	13.2±3.5	12.9±3.2	0.384
Nitrate (mg kg^−1^ soil)	46.8±12.1	31.6±7.3	0.402	24.5±3.9	22.9±7.0	0.866
Ammonium (mg kg^−1^ soil)	2.8±0.3	2.9±0.3	0.105	2.5±0.2	2.7±0.3	0.316
Phosphorus (mg kg^−1^ soil)	60.9±13.3	64.5±7.7	0.652	60.1±13.5	72.1±10.4	0.173
Sulfur (mg kg^−1^ soil)	134±30	119±38	0.76	119±39.4	55.7±14.8	0.140
Boron (mg kg^−1^ soil)	0.88±0.23	0.74±0.25	0.043	0.71±0.19	0.75±0.30	0.441
Zinc (mg kg^−1^ soil)	2.88±0.37	1.97±0.12	0.048	2.42±0.37	1.81±0.24	0.097
Manganese (mg kg^−1^ soil)	4.52±0.50	7.64±2.01	0.217	3.13±0.37	3.68±0.47	0.196
Copper (mg kg^−1^ soil)	1.37±0.31	1.17±0.25	0.216	1.40±0.31	1.23±0.29	0.291
Iron (mg kg^−1^ soil)	28.6±3.9	26.8±5.0	0.064	26.4±3.4	31.4±5.4	0.203
Potassium (cmol kg^−1^ soil)	0.6±0.1	0.5±0.1	0.194	0.6±0.1	0.5±0.1	0.230
Calcium (cmol kg^−1^ soil)	10.7±2.3	9.7±2.1	0.165	10.3±2.5	9.6±2.2	0.519
Magnesium (cmol kg^−1^ soil)	4.1±1.1	4.2±1.3	0.722	3.9±1.2	4.20±1.3	0.695
Sodium (cmol kg^−1^ soil)	0.4±0.1	0.3±0.1	0.001	0.3±0.04	0.3±0.04	0.858
Total bases (cmol (+) kg^−1^)	15.8±3.5	14.7±3.3	0.244	15.1±3.7	14.6±3.5	0.841
pH	7.05±0.11	7.09±0.16	0.953	7.16±0.10	7.09±0.17	0.694
Buffer capacity pH	7.51±0.02	7.51±0.03	0.908	7.53±0.02	7.52±0.03	0.789
EC (mmhos cm^−1^)	2.72±0.34	2.18±0.39	0.071	2.13±0.37	1.50±0.22	0.306
Total carbon (g kg^−1^ soil)	10.04±0.15	8.25±0.12	0.036	9.43±0.17	7.71±0.13	0.034
Total nitrogen (g kg^−1^ soil)	0.867±0.014	0.666±0.010	0.009	0.783±0.015	0.625±0.012	0.010
Readily mineralizable carbon (µg MinC g^−1^ soil)	17.7±1.1	14.1±1.2	0.009	14.9±1.6	11.2±1.2	0.019
Microbial biomass (µg MicC g^−1^ soil)*	249±22.5	96±6.8	0.000	211±20.5	101±12.1	0.042
MicC (% of total carbon)*	2.21±0.13	1.33±0.26	0.005	2.16±0.31	1.54±0.37	0.041
MicC MinC^−1^ *	16.0±1.8	8.6±0.6	0.004	16.8±3.1	9.3±0.5	0.049
Basal respiration (µg CO_2_-C g^−1^ soil h^−1^)*	0.472±0.055	0.354±0.032	0.009	0.731±0.186	0.348±0.111	0.009
Dehydrogenase (µg TPF g^−1^ soil)	1.38±0.21	0.65±0.05	0.000	0.89±0.14	0.52±0.05	0.000
Acid phosphatase (µg p-nitrophenol g^−1^ soil)	121.5±14.1	58.2±5.6	0.009	104.7±37.4†	53.1±9.1†	0.039
Alkaline phosphatase (µg p-nitrophenol g^−1^ soil)	122.3±13.0	55.6±8.5	0.002	84.4±17.0†	47.0±13.0†	0.262
qCO_2_ (ug CO_2_-C h^−1^ mg^−1^ MicC)*	1.9±0.18	3.7±0.33	0.003	3.5±0.73	3.4±0.67	0.838
Protease native (µg amino acid-N g^−1^ soil h^−1^)	2.41±0.29	2.81±0.36	0.446	2.08±0.29†	1.25±0.35†	0.107
Protease potential (µg amino acid-N g^−1^ soil h^−1^)	4.06±0.65	3.49±0.32	0.369	3.21±0.25†	2.78±0.31†	0.150
Mycorrhizae total colonized root length (mm)*	122±11	104±10	0.164	–	–	–

Soil samples were taken in June 2004 and June 2005, except where noted. Means and standard errors of soil properties for individual years are listed in Tables S5A and B.

*Measured in June 2005 only.

† Measured in June 2004 only.

Organically managed surface soils also supported significantly greater microbial biomass (159.4% more), microbial carbon as a percent of total carbon (66.2% greater), readily mineralizable carbon (25.5% more), and microbial carbon to mineralizable carbon ratio (86.0% greater) (Table 6). These indicate larger pools of total, labile, and microbial biomass C and a higher proportion of soil total and labile C as microbial biomass. All measures of microbial activity were significantly greater in the organically farmed soils, including microbial respiration (33.3% more), dehydrogenase (112.3% more), acid phosphatase (98.9% more), and alkaline phosphatase (121.5% more). The organically farmed soils had a significantly lower qCO_2_ metabolic quotient, indicating that the microbial biomass in the organically farmed soils was 94.7% more efficient or under less stress than in the conventionally farmed soils [40]. These same differences, except for qCO_2_, alkaline phosphatase, iron, boron, and sodium, were also observed in soils from the bottom of the mounds (20–30 cm depth).

To quantify soil microbial gene presence and diversity, we used a gene array termed GeoChip containing more than 24,000 oligonucleotide (50-mer) probes and covering 10,000 genes involved in nitrogen, carbon, sulfur, and phosphorus transformations and cycling, metal reduction and resistance, and organic xenobiotic degradation [27], [41]. Microarray genes were analyzed both individually and within functional groups in soil samples from organic and conventional strawberry fields [42]. A functional group is a group of genes involved in a certain function or biogeochemical process in the soil. In this study, the following 11 functional groups were targeted: nitrogen fixation, nitrification, denitrification, sulfite reduction, pesticide degradation, other organic xenobiotic degradation, metal reduction and resistance, and genes for the enzyme classes dehydrogenase, urease, cellulase, and chitinase.

**Figure 2:**
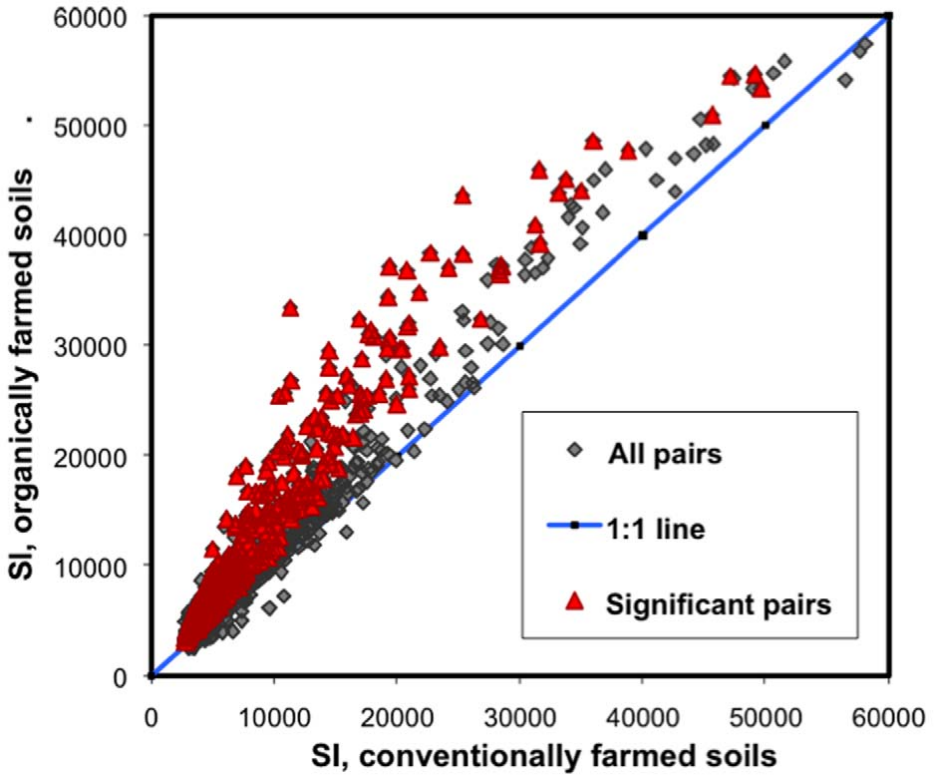
A scatter plot of signal intensities (SIs) of 1711 individual genes on GeoChip microarrays. Each of the 1711 data points represents average gene SI from eight organically farmed soils against eight matched conventionally farmed soils. The SIs of more than 32% (553) of 1711 individual genes detected were significantly higher in organically managed soils, while not one was significantly higher in conventionally managed soils.

Mean DNA microarray signal intensity of total detected genes was significantly greater in organically managed soils than in conventionally managed soils (Table 7) [42]. Similarly, mean signal intensities for the 11 gene functional groups were all significantly greater in organically managed soils (Table 7). The signal intensities of more than 32% (553) of 1711 individual genes detected were significantly higher in organically managed soils, while not one was significantly higher in conventionally managed soils (Figure 2). Signal intensity is correlated with gene copy number and dependent on DNA labelling efficiency [26]. Mean labelling efficiency of DNA from organic and conventional soils was similar (1.23 and 1.25 µmol Cy 5/µl DNA solution, respectively, *P* = 0.78), demonstrating that the detected differences were not introduced by differing labelling efficiencies and that functional genes and likely the organisms that carry them were more abundant in organically managed soils.

**Table 7:** Soil DNA microarray signal intensity and diversity (mean ± standard error) of total detected genes and gene functional and organism groups from organic (ORG) and conventional (CON) strawberry farms (n = 8).

Soil Functional Group or Organism Group	Signal Intensity (10^3^)			Diversity (Simpson's Reciprocal Index)		
	ORG	CON	*P* Value	ORG	CON	*P* Value
Total detected genes	13479±874	9350±1003	0.008	656±31	504±34	0.015
N fixation	744±59	547±76	0.018	44±1	38±2	0.034
Nitrification	262±12	201±13	0.004	7±0.3	6±0.3	0.012
Denitrification	552±46	405±59	0.029	33±2	26±3	0.010
Sulfite reduction	529±36	368±40	0.009	37±1	31±1	0.004
Pesticide degradation	1970±131	1322±143	0.006	104±4	81±4	0.004
Other organic xeno-biotic degradation	3999±253	2819±296	0.008	193±10	146±11	0.012
Metal reduction and resistance	2580±164	1750±164	0.008	112±5	84±5	0.010
Dehydrogenase	171±9	118±11	0.004	7±0.3	6±0.3	0.245
Urease	621±48	394±59	0.008	36±3	28±3	0.031
Cellulase	819±60	569±65	0.012	51±2	41±2	0.012
Chitinase	347±25	240±23	0.016	16±1	12±0.7	0.024
Fungi	164±9	108±10	0.003	17±4	13±3	0.025
Prokaryotes	12818±837	9088±964	0.008	624±29	487±33	0.011
Fungi/Prokaryotes Ratio	0.013±0.000	0.012±0.000	0.323	0.027±0.007	0.027±0.006	0.343

Functional groups from Reeve et al. [42].

Organically managed soils exhibited significantly more endemic genes (*P*<0.0001); more specifically, 233 genes were detected only in the organically managed soils and 2 genes were detected only in conventionally farmed soils (Table S1). Genetic diversity was also significantly greater in organically managed soils across all detected genes (total) and for 10 of the 11 functional groups (Table 7). Greater diversity within a functional group may simply be redundant, particularly at high levels of diversity [43]. Conversely, greater diversity may help support the resulting ecosystem function or biogeochemical process in a broader range of environmental conditions [44] or in changing environments [45]. Our findings of greater enzyme activities in organically managed soils indicate a greater functional capacity. Greater functional gene abundance in organically managed soils indicates a larger functional population. Greater functional gene diversity in organically managed soils suggests that organic systems may also support more stable or resilient ecosystem functioning.

Some of the 11 functional groups addressed on the GeoChip are purely prokaryotic functions (e.g., nitrogen fixation, nitrification, and denitrification), while others are characteristics of both prokaryotes and eukaryotes (mainly fungi). To ensure that neither group biased the signal intensity and diversity results for either the organic or conventional farming systems, we separated out fungal- and prokaryotic-derived genes into their respective groups and calculated the ratio of fungi to prokaryotes for gene signal intensity and diversity in the two agroecosystems. Not only are the fungi numbers higher for both signal intensity and diversity in the organic agroecosystems, but so are the prokaryote numbers, too (Table 7). However, ratios of fungi/prokaryotes for either signal intensity or diversity are similar for the two farming systems, which dispels the concern that the data presented selectively favored prokaryotes over eukaryotes in either the organic or conventional agroecosystems.

The large differences in soil microbial properties and soil functional gene abundance and diversity between the organically and conventionally farmed soils are most likely due to a combination of factors: chemical fumigation with methyl bromide of the conventionally farmed soils, lack of synthetic pesticide use on the organic fields, and double the application rates of compost to the organic fields compared to the conventional fields (Table S2). A number of studies have documented changes in microbial diversity due to fumigants and pesticides [46], [47], [48], although the majority of changes were short-term, with microbial populations generally returning to normal after a few weeks or months. Many of these studies were conducted using simulated agricultural fumigation in a laboratory, and nearly all used a single fumigation event with no regard to past history of fumigation or pesticide use on the studied soil. Two- to three-year field studies with annual fumigation have shown methyl bromide to alter some microbial properties and enzymatic functions but the effects were inconsistent [49], [50].

Our study, in which soil samples were taken about 5 to 6 months after fumigation, was conducted on organic and conventional fields with longer histories (at least 5 years) of both organic and conventional (with fumigation) management, likely contributing to the detection of some persistent effects on the microbial population. The organic fields also received 20.2–24.6 Mg compost ha^−1^, almost twice the rate of the conventional fields at 11.2–13.4 Mg compost ha^−1^ (Table S2). Mäder et al. [23] found that soil amendment with animal manures in organic farming systems increased microbial biomass and enzyme activity and altered the structure of the microbial community. Crop rotations likely played a minor role in the differences in soil properties in our study because rotations were similar for the organic and conventional agroecosystems; that is, the organic and conventional farms used two-year rotations, in which strawberries were followed by broccoli, lettuce, or a cover crop in the second year.

In summary, the organic strawberries and their soils were of higher quality compared to their conventional counterparts. Specifically, the organic strawberries, while having lower concentrations of phosphorus and potassium, had higher antioxidant activity and concentrations of ascorbic acid and phenolic compounds, longer shelf life, greater dry matter, and, for ‘Diamante’, better taste and appearance. The organically farmed soils had more carbon and nitrogen, greater microbial biomass and activity, and greater functional gene abundance and diversity. This study demonstrates that soil DNA analyses using microarray technology can be used as an additional measurement of soil quality. Our sustainability study also demonstrates the benefits of using an interdisciplinary methodology that comprehensively and quantitatively compares numerous indices of fruit and soil quality from multiple, commercial organic and conventional farms, multiple varieties and soils, and multiple sampling times.

## Methods

### Study Area

Thirteen pairs of side-by-side commercial organic and conventional strawberry farm fields were selected in the Watsonville area, the dominant strawberry growing region of California, USA. In turn, California is the leading producer in the U.S., accounting for 87% of the nation's strawberry production (29). The Watsonville area annually grows strawberries on about 5,000 hectares, accounting for about 40% of the strawberry acreage in the state (29).

The selection of 13 field pairs (5 in 2004 and 8 in 2005) from commercial strawberry farms was made on the basis of grower interviews and on-farm field examinations to ensure that all soil-forming factors, except management, were the same for each field pair [21]. Each field pair consisted of two side-by-side fields, one organic and one conventional. Fields chosen in each pair had the same microclimate, soil profile, soil type, soil classification, and strawberry variety (Table S3).

Strawberry field pairs in 2004 were different from those in 2005 because both organic and conventional farmers grew strawberries in alternate years using a similar two-year rotation. More specifically, all farmers in the study grew strawberries on constructed, 30-cm high mounded rows covered with plastic mulch for only one year, preceded by a different crop, such as broccoli, lettuce, or a cover crop, grown on flat ground (without mounds) the previous year. Growing strawberries as annuals using this “raised-bed plasticulture” system is typical of organic and conventional strawberry growers in California [51]. Growers in the study transplanted strawberry crowns in November, with strawberry plants starting to produce fruit in mid-March and continuing to produce fruit to mid- or end-October.

The organic fields had been certified organic (USDA) for at least 5 years, providing sufficient time for the organic farming practices to influence soil properties. The organic fields relied only on organically certified fertilizers and pesticides and no soil fumigation (Table S2). Both organic and conventional farms applied compost, with the organic strawberry systems using 20.2–24.6 Mg compost ha^−1^ and the conventional systems using 11.2–13.4 Mg compost ha^−1^ (Table S2). These high rates of compost additions along with organic fertilizer amendments permitted strawberries to be grown in the two-year rotation described above. The conventional farms also had been managed conventionally for at least 5 years and included the use of inorganic and organic fertilizers, synthetic pesticides, and soil fumigation (methyl bromide with or without chloropicrin) (Table S2). Conventional strawberry growers in California typically rely on methyl bromide (with or without chloropicrin) as an extremely effective broad-spectrum, pre-plant biocide to kill soil-borne diseases (including fungi and bacteria), nematodes, soil-dwelling insects, weeds, weed seeds, and underground plant parts [51].

### Strawberry Sampling and Analyses

Strawberry (*Fragaria x ananassa* Duch.) varieties grown on the study farms included ‘Diamante’ and ‘San Juan’ in 2004 and ‘Diamante’, ‘San Juan’, and ‘Lanai’ in 2005. Strawberry fruit were collected from each of 13 pairs of organic and conventional strawberry farm fields in June and September 2004 and April, June, and September 2005. Commercial pickers harvested and packed ripe fruit in plastic “clamshells,” just as they would be sold in retail markets. All strawberries were picked at random from within 8 to 15 rows and were always a minimum of 20 m from the boundary within each field pair to avoid edge effects. Within hours, packed fruit were transported to a refrigerated storage facility until shipped on commercial refrigerated trucks from Watsonville, CA to distribution centers in Seattle or Spokane, WA. These samples were transported under cool conditions to Washington State University in Pullman, WA, where they were immediately placed in refrigerated storage.

A subsample of the collected fruit, as well as leaf samples, taken in April 2005 and June 2004 and 2005, were sent to Soiltest Farm Consultants Inc. in Moses Lake, WA, where fruit and leaf samples were analyzed for N, P, K, Ca, Mg, B, and Zn, plus S, Mn, Cu, and Fe for leaf samples only, according to standard methods [52]. All other strawberry analyses, including shelf life, fresh weight, dry matter, firmness, color, total antioxidant activity, ascorbic acid, total phenolics and anthocyanins, and specific polyphenols, were conducted at Washington State University, Pullman, WA. Since our study focused on the nutritional differences of fresh strawberries as consumed, we expressed our nutritional composition data on the basis of how the product would be eaten, or fresh weight, which is the standard for the USDA National Nutrient Database for Standard Reference [36], FAO's International Network of Food Data Systems (INFOODS) [53], and the United Kingdom's Food Standards Agency [54].

Strawberry fruit from each field and sampling time were subsampled for fresh analysis within two days of receipt at Washington State University in Pullman, WA, with another subsample stored at −80°C for later biochemical analysis. On each sample time, 20 fruit from each field were weighed fresh, ten of which were dried in an oven at 80°C and reweighed to determine dry weight, while the other 10 fruit were left at room temperature (∼20°C) for two days and reweighed to estimate weight loss. Fruit firmness was measured as maximum penetration force (N) on opposite sides of another 25 fruit from each field with an automated penetrometer (Model GS-20 Fruit Texture Analyzer, Güss Manufacturing Ltd., Strand, South Africa) fitted with a 5-mm diameter convex cylinder set to a trigger threshold of 1.11 N and 6-mm depth. On each of these fruit, two external (on opposing shoulders) and two internal (adjacent to central cavity) color measurements (Model CR-300 Chroma Meter, Minolta Camera Co., Ltd., Ramsey, NJ) were taken using the L*a*b* color space expressed as lightness (L*), chroma (C*, [(a*)^2^+(b*)^2^]^1/2^) and hue angle (h_ab_, tan^−1^[b*/a*]) [55]. Soluble solids content (%) in the homogenate from these berries was measured in triplicate with a digital refractometer (Model PR-101, Atago Co., Ltd., Tokyo, Japan), as were pH and titratable acidity (citric acid equivalents), using an automated titrator (Schott Titroline *easy*, Schott-Geräte GambH, Mainz, Germany) with 0.1 N KOH (pH 12.8). The ratio of soluble solids to titratable acidity was also calculated.

In order to estimate the susceptibility of the strawberries to fungal rots, a subsample of 72 fresh fruit from each field and sampling time were placed in individual cells (6.7 cm×5.9 cm×5.7 cm deep) of plastic greenhouse inserts (Model IKN3601, ITML Traditional Series Inserts, Hummert International, Earth City, MO). Two inserts with 36 berries in each were placed in trays with dampened paper to maintain a saturated atmosphere and in sealed, black plastic bags. For both sample months in 2004, fruit were incubated at 15.5°C for 9–10 days, with the number of rotted berries counted each day. All rotted fruit were removed from their cells until all fruit had rotted. The principal fungal rot observed on the berries was gray mold (*Botrytis cinerea*).

For analysis of antioxidant activity, ascorbic acid, total phenolics, anthocyanins, and total and reducing sugars, chemicals and enzymes were purchased from Sigma-Aldrich Corp. (St. Louis, MO), unless otherwise noted. Spectrophotometric measurements were made using a UV-visible spectrophotometer (Model HP8453, Hewlett-Packard Co., Palo Alto, CA) with UV-Visible ChemStation software [Rev. A.08.03(71), Agilent Technologies, Inc., Santa Clara, CA]. All solutions were made up using ultrapure water (NANOpure DIamond Analytical, Barnstead International, Dubuque, IO). Centrifugation was performed in a Eppendorf 5417 R microcentrifuge (Engelsdorf, Germany). There were 3–5 separate replicates of pooled tissue from a minimum of five fruit analyzed in each biochemical assay, with duplicate instrument measurements made on each replicate. Outlying data were discarded and the tissue reanalyzed.

Antioxidant activity of hydrophilic and lipophilic fractions [56] in the berries was measured by the end-point 2,2′-azino-bis-(3-ethylbenzthiazoline-6-sulfonic acid) (ABTS)/hydrogen peroxide/peroxidase (Horseradish peroxidase, HRP, Type VI-A) method of Cano *et al.* [57], with modifications. Specifically, 100 mg powdered, frozen berry tissue was extracted in 700 mL 50 mM MES (pH 6.0) and 700 mL ethyl acetate, vortexed for 30 sec, and centrifuged at 13 K rpm for 10 min at 4°C. The organic (top) and aqueous (bottom) phases were separated with a pipette for measurement of lipophilic and hydrophilic antioxidant activities (LAA and HAA, respectively). For both fractions, 40 mL 1 mM H_2_O_2_, 100 mL 15 mM ABTS, and 10 mL 3.3 U mL^−1^ HRP were placed in 1 mL quartz cuvettes and gently shaken for 10 sec, after which 830 mL 50 mM phosphate buffer (pH 7.4) was added and mixed with a stir paddle. Absorbance was monitored at 734 nm on a UV-visible spectrophotometer until stable (<10 sec), and then 20 mL (for HAA) or 40 mL (for LAA) extract was added, mixed with a stir paddle, and monitored at 734 nm until absorbance reached a minimum. HAA and LAA were calculated from the absorbance difference and expressed on the basis of Trolox equivalents from standard curves of 5 mM Trolox diluted in 50 mM MES buffer (pH 6.0) or 100% (v/v) ethyl acetate, respectively, and measured as described for the samples. HAA and LAA were summed to estimate total antioxidant activity (TAA).

Total ascorbic acid (reduced AsA plus dehydroascorbic acid, DHA) in the berries was measured as originally described by Foyer *et al.* [58] and modified by Andrews *et al.* [59]. Specifically, 200 mg powdered, frozen berry tissue was extracted in 1.5 mL ice-cold 5 M HClO4 by grinding with liquid nitrogen in a mortar and pestle. Samples were transferred into 2 mL brown, microcentrifuge tubes, vortexed for 30 sec, and centrifuged at 13 K rpm for 10 min at 4°C. Into two, 400 mL aliquots of supernatant from each extract, 200 mL 0.1 M HEPES-KOH buffer (pH 7.0) were added and mixed, followed by 20–30 mL 5 M K_2_CO_3_ to reach pH 4–5. Following centrifugation, 200 mL supernatant was reduced by adding 31.8 mL 1 M DL-dithiothreitol (DTT) in 400 mL 100 mM phosphate buffer (pH 5.6), gently shaking and incubating on ice for 5 min. Absorbance of 100 mL of reduced extract in 396 mL 100 mM phosphate buffer (pH 5.6) in a blackened, 0.5 mL quartz cuvette was monitored at 265 nm on a UV-visible spectrophotometer until stable (<10 sec), and then 4 mL 1 U mL^−1^ ascorbate oxidase (from *Cucurbita*) was added, mixed with a stir paddle, and monitored at 265 nm until absorbance reached a minimum. Concentration of total ascorbic acid was calculated from the absorbance difference and standard curves of 5.25 mM dehydro-L-(+)-ascorbic acid dimer reduced with 265 mL 1 M DTT in 400 mL 100 mM phosphate buffer (pH 5.6) and monitored at 265 nm as described for the samples.

Total phenolic compounds in the berries were measured with the Folin-Ciocalteu (F–C) phenol reagent (2 N) according to revised methods of Singleton *et al.* [60]. Specifically, to 200 mg powdered, frozen berry tissue, 1 mL 80% (v/v) methanol was added in microcentrifuge tubes. Samples were vortexed, allowed to extract 1 h at room temperature and then overnight at −20°C, followed by centrifugation at 14 K rpm for 20 min at 4°C. The supernatants were removed and extraction of the pellet was repeated 2× as described, with supernatants combined after each extraction and then made up to 4 mL with 80% (v/v) methanol after the final extraction. Total phenolic compounds were assayed by adding 400 mL sample extract into two 15-mL tubes containing 600 mL 80% (v/v) methanol, 5 mL 10% (v/v) F-C reagent, and either 4 mL saturated Na_2_CO_3_ (75 g L^−1^) or 4 mL water. Tubes were thoroughly mixed and incubated at room temperature for 2 h. One-milliliter aliquots from the sample tubes containing Na_2_CO_3_ or water were added to 1.5 mL plastic cuvettes and the absorbance of each was measured at 760 nm in a UV-visible spectrophotometer. Concentration of phenolic compounds was determined by subtracting absorbance of samples containing Na_2_CO_3_ from those not containing Na_2_CO_3_, quantified as gallic acid (3,4,5-trihydroxybenzoic acid) equivalents from standard curves.

For anthocyanins, 0.5 g of powdered, frozen berry tissue was extracted in 1 mL 1% (v/v) HCl-methanol. After storage for 24 h at −20°C, sample tubes were centrifuged at 14 K rpm for 10 min at 4°C. Extraction with HCl-methanol was repeated 2×. Following centrifugation on day four, supernatants were decanted into 15 mL plastic tubes and made up to 3-mL volumes with HCl-methanol. Anthocyanin concentrations were determined by measuring absorbance of 250 mL extract in 750 mL 1% (v/v) HCl-methanol in 1.4 mL quartz cuvettes at 515 nm with a UV-visible spectrophotometer [61], expressed as pelargonidyn-3-glucoside equivalents using E_molar_ = 3.6×10^6^ M^−1^ m^−1^.

Specific polyphenolic compounds were extracted by grinding 0.1 g frozen, powdered fruit tissue in 1.5 mL pure methanol. Concentrations of aglycones of ellagic acid, quercetin, kaempferol, phloretin, and naringenin enantiomers were determined, as well as the total aglycone plus glycoside polyphenols, following enzymatic hydrolysis with β-glucuronidasefrom *Helix pomatia* (Type HP-2) [62], [63]. Extracts (150 mL), with daidzein as internal standard (IS), were injected into a HPLC system (Shimadzu, Kyoto, Japan), consisting of LC-10AT VP pump, SIL-10AF auto injector, SCL-10A system controller. Polyphenols were separated isocratically with a mobile phase of acetonitrile∶water∶phosphoric acid (v/v/v 42∶58∶0.01 at 0.6 mL min^−1^ for ellagic acid, quercetin, kaempferol, and phloretin; 30∶70∶0.04 at 0.4 mL min^−1^ for naringenin enantiomers) at 25°C on chiral stationary phase amylose- or cellulose-coated columns (Chiralcel AD-RH for ellagic acid, quercetin, kaempferol, and phloretin and Chiralcel OD-RH for naringenin enantiomers, with 5 mm particle size and 150 mm×4.5 mm ID; Chiral Technologies Inc., Exton, PA, USA), and detected at 370 nm (for ellagic acid, quercetin, kaempferol, and phloretin) and 292 nm (for naringenin enantiomers) on a Shimadzu SPD-M10A VP diode array spectrophotometer. Data collection and peak integration were carried out using Shimadzu EZStart 7.1.1 SP1 software. Individual polyphenols were quantified based on standard curves constructed using peak area ratio (PAR = PA_polyphenol_/PA_IS_) against the concentration of the standards. Best laboratory practices during sample analysis followed guidance, based upon the International Conference on Harmonisation (http://www.ich.org/), for the quantitative analysis of polyphenolic compounds using a validated assay and commercially available standards, with all samples run in duplicate with appropriate quality controls [64], [65].

Reducing and total sugars were measured by the Nelson-Somogyi micro-colorimetric method [66], with modifications. Specifically, 0.1 g frozen berry homogenate was extracted in 1.5 mL pure methanol for 30 min at room temperature, after vortexing for 30 sec. Total sugars were obtained by adding 150 mL 0.1 M HCl to duplicate tissue samples and allowing hydrolysis of sugars for 10 min prior to methanol extraction. Samples were then centrifuged at 14 K rpm for 10 min. Supernatants (0.2 mL), diluted with 0.8 mL water, were mixed with 1 mL copper-sulfate reagent in glass tubes with stoppers and incubated for 10 min in a boiling water bath. After cooling for 5 min, 1 mL arsenomolybdate reagent was added and mixed. Volumes were adjusted to 10 or 25 mL with deionized water, depending on color density. Concentrations of reducing and total sugars were determined by measuring absorbance at 520 nm with a UV-visible spectrophotometer and quantified by standard curves of glucose made from stock 1% (w/v) glucose solution in saturated benzoic acid.

### Sensory Analyses

We also conducted consumer-sensory analyses of strawberries, including flavor, sweetness, appearance, juiciness, tartness, and overall acceptance. Strawberries were evaluated by consumer-sensory panels at four different sampling dates (20 panelists per field pair in Sept 2004 and 25 panelists per field pair in April, June, and Sept 2005) at WSU's Food Science and Human Nutrition Sensory Laboratory. Panelists were recruited using advertising from the Washington State University community based on their availability. A minimum amount of information on the nature of the study was provided in order to reduce potential bias. All participants signed an Informed Consent Form per project approval by the WSU Institutional Review Board.

Each panelist completed a demographic questionnaire prior to the start of the panel. Fifty-eight percent of the panelists were females. The age distribution of the panelists was 31% 18–25 years old, 41% 26–35 years old, 10% 36–45 years old, 13% 46–55 years old, and <5% over 55 years old. Over 70% of the panelists ate fresh strawberries every two weeks to every month, with 19% eating fresh strawberries every week. The majority of panelists (59%) preferred fresh strawberries that tasted more sweet than tart and another 36% preferred them at least equally sweet and tart. Less than 5% of the panelists preferred them more tart than sweet or had no preference.

Each consumer received organic and conventional berries from two, matched field pairs. Consumers were presented with two strawberry halves from two individual strawberries. Each sample was presented in a monadic, randomized serving order with assigned three-digit codes. Each panelist was also provided with deionized, filtered water and unsalted crackers for cleansing the palate between samples.

Consumers evaluated each strawberry sample for overall acceptance, as well as perceived intensity of flavor, juiciness, sweetness, and sourness using a discrete 9-point, bipolar hedonic/intensity scale, where 1 = dislike extremely/extremely low intensity and 9 = like extremely/extremely high intensity, according to ISO standards for quantitative response scales [67]. These evaluations were completed under red lights to disguise color differences between the samples. Following the taste/flavor evaluations, the lights were changed to white lights and panelists evaluated the strawberries for acceptance of appearance using the same 9-point scale.

### Statistical Analyses of Strawberry Data

Mixed model analyses of variance were used to test for differences in response variable means, except where noted, due to varieties (‘Diamante’, ‘Lanai’, and ‘San Juan’), treatments (organic and conventional), and months (April, June, and September). A split plot model pooled over two years was selected with variety as the whole plot factor, treatment as the subplot factor, and month as a repeated measure (SAS Proc Mixed, SAS Institute, 1999). Transformations were used to improve normality and homogeneity of variances where necessary. When data were transformed, LS means were reported in original units. When significant interactions were identified, differences in simple effect means were identified using Fisher's least significant differences. The same mixed model analysis of variance was applied to examine sensory data by using the average panel score for each attribute. The Kaplan-Meier (Product Limit) method was used to model the survival function and estimate mean survival time, that is, days to rotting (SAS Proc Lifetest, SAS Institute, 1999). The generalized Savage (Log-Rank) test for equality of survival functions was used to test for differences in time to rotting for organic versus conventional conditions [68].

### Soil Sampling and Analyses

Soils were sampled from 30-cm raised mounds at 0–10 cm and 20–30 cm depths in June 2004 and June 2005 and at 0–10 cm in April 2005. All samples were a composite of 10–15 subsamples taken at random from within 8 to 15 rows and were always a minimum of 20 m from the boundary within each field pair to avoid edge effects. Samples from the June sampling dates were shipped for chemical analyses to Soiltest Farm Consultants and for biological analyses to Washington State University by overnight mail. Samples from the April 2005 sampling were shipped to Oak Ridge National Lab for microarray analyses and stored at −20°C. Raw microarray data are in Data S1, S2, S3, and S4 and can also be found at <http://www.ornl.gov/~cys/MMEresearch.html>.

At Soiltest Farm Consultants, soil samples were passed through a 2-mm sieve, stored at 4°C, and then analyzed for the following properties according to recommended soil-testing methods by Gavlak et al. [52]: Nitrate-nitrogen (N) was measured with the chromotropic acid method; ammonium-N was measured with the salicylate method; Olsen phosphorus was measured; DTPA-Sorpitol extractable sulfur, boron, zinc, manganese, copper, and iron were measured; Soil pH and electrical conductivity were measured in a 1∶1 w/v water saturated paste; SMP soil buffer pH was measured; NH_4_OAc extractable potassium, calcium, magnesium, and sodium were measured; total bases were calculated by summation of extractable bases; and particle size (percentage sand, silt, and clay) was analyzed by the hydrometer method.

At Washington State University, we analyzed total C and N by combustion using a Leco CNS 2000 (Leco Corporation, St. Joseph, MI). Readily mineralizable carbon (MinC), basal microbial respiration, and active microbial biomass (MicC) by substrate-induced respiration were measured according to Anderson and Domsch [69]. Ten grams of wet weight soil were brought to 12, 18, and 26% moisture content (−0.033 MPa), depending on soil type, and incubated at 24°C for 10 days. Total CO_2_ released after 10 days was considered MinC. Vials were recapped for 2 h and the hourly rate measured for microbial respiration. For MicC, 0.5 mL of 12 g L^−1^ aqueous solution of glucose was added to the same soil samples and rested for 1 h before being recapped for 2 h. Carbon dioxide was measured in the headspace using a Shimadzu GC model GC -17A (Shimadzu Scientific Instruments, Columbia, MD), with a thermal conductivity detector and a 168 mm HaySep 100/120 column. From these microbial properties, we calculated the metabolic quotient, qCO_2_ (basal respiration/MicC), and the two ratios, MicC/MinC and MicC as a percent of total C. Dehydrogenase enzyme activity was measured using 2.5 g dry weight soil and acid and alkaline phosphatase enzyme activities were measured using 1 g dry weight soil as described by Tabatabai [70]. These enzyme reactions were measured using a Bio-Tek microplate reader model EL311s (Bio-Tek Instruments, Winooski, VT). Both native and potential protease enzyme activities were measured using 1 g dry weight soil according to Ladd and Butler [71] and measured on a Perkin Elmer Lambda 2 UV/VIS spectrometer (PerkinElmer Life And Analytical Sciences, Inc, Waltham, MA) at 700 nm with tyrosine standards. Native protease represents activity without the addition of casein substrate and potential protease represents the activity with the addition of substrate. Arbuscular mycorrhizae were stained with trypan blue and total and colonized roots estimated using the gridline intersection method [72].

### Statistical Analyses of Soils Data

June comparisons of soil under organic and conventional management were analyzed as a randomized complete block design with split plot. Year served as whole plot and treatment as subplot. The two depth intervals were analyzed separately. All statistics were analyzed using the SAS system for Windows version 9.1 ANOVA and LS means (SAS Institute, 1999). Data were checked for model assumptions and transformed as necessary. When data were transformed, LS means were reported in original units.

### Microarray Analyses

Soil community DNA was extracted using an SDS-based method [73]. A total of 10 g soil from each organic field and 20 g soil from each conventional field (due to low yields of DNA) were used. DNA was purified using a Wizard PCR cleanup system (Promega, Madison, WI). The cleaned pellet was washed in 500 µl ethanol (70%) before being resuspended in 20 µl 10 mM Tris (pH 8.0). Microarray slides were constructed according to methods described previously [26]. We used a comprehensive functional gene array, termed GeoChip, containing more than 24,000 oligonucleotide (50-mer) probes and covering 10,000 genes involved in nitrogen, carbon, sulfur, and phosphorus transformations and cycling, metal reduction and resistance, and organic xenobiotic degradation [41]. Microarray genes were analyzed both individually and as 11 functional groups: nitrification, denitrification, nitrogen fixation, sulfite reduction, pesticide degradation, other organic xenobiotic degradation, metal reduction and resistance, dehydrogenase, urease, cellulase, and chitinase.

Thirty to 150 ng purified DNA from each soil was randomly amplified using rolling circle PCR with a GenomiPhi DNA amplification kit (GE Healthcare, Piscataway, NJ) [74]. The amplification product was fluorescently labeled with Cy5 dye with an extended 6 h incubation time and applied directly to the microarray. Mean labeling efficiency per treatment was calculated to ensure no overall bias. Slides and all solutions were kept at 60°C during assembly to minimize cross contamination. Hybridizations were carried out at 50°C with 50% formamide [26]. After hybridization, the slides were immediately placed in wash solution 1 (1×SSC and 0.1% SDS) to remove the cover slip and washed by gentle shaking in solution 1, 2 times for 5 min each; then washed in solution 2 (0.1×SSC and 0.1% SDS), 2 times for 10 min each; and finally in solution 3 (0.1×SSC), 4 times for 1 min each. Arrays were then dried using compressed air. All arrays were run in triplicate. The microarrays were scanned using a ScanArray 5000 analysis system (Perkin-Elmer, Wellesley, MA) [26].

### Microarray Data Processing and Analyses

Microarray slide images were converted to TIFF files and hybridized DNA quantified using ImaGene software 6.0 (Biodiscovery Inc., Los Angeles, CA) [26]. The signal-to-noise ratio (SNR) of each probe on each slide was calculated as follows: SNR =  (signal intensity – local background) (standard deviation of slide background)^−1^. Background refers to the local background intensity, while the standard deviation was calculated across the whole slide. Signal intensity data for any gene was removed unless it appeared with SNR>2 on at least two of three replicate array hybridizations. When this condition was met, individual SNR values<2 were retained in order to maintain a continuous data set for statistical analysis. The array included multiple probes for some genes; here the strongest signal was retained and weaker ones deleted. After screening for adequate SNR, signal intensity values were then used as the data for sample comparison. Signal intensity values were normalized by averaging across technical replicates and imported into SAS system for Windows version 9.1 ANOVA (SAS Institute, Cary, NC) for analysis.

Data were analyzed using a randomized complete block design, with field pair as block. Average signal intensity for each of the 1711 detected genes, sum of signal intensities for all 1711 detected genes, and sum of signal intensities for each of the 11 functional groups from the eight organically farmed soils and the eight matched conventionally farmed soils were analyzed by paired t-tests. Gene diversity was calculated overall and for each functional group using a modified version of Simpson's Reciprocal Index [D = 1/[∑n(n−1)/N(N−1)], where n =  signal intensity of a single gene with an SNR>2 and N =  sum of all signal intensities with an SNR>2 on the entire slide]. Diversity values were then analyzed by a paired t-test. Detected endemic genes were counted based on treatment means. Proportion comparison z tests were used to compare proportion of detected endemic genes in each management system.

## Supporting Information

Table S1 Two gene sequences endemic to conventionally managed field soils and 233 sequences endemic to organically managed field soils, and the organisms from which probes were designed.
(0.15 MB DOC)

Table S2 Agrichemical inputs (insecticides, fungicides, herbicides, molluscides, adjuvants, fumigants, and fertilizers) applied to 26 strawberry fields during the 2004 and 2005 growing seasons.
(0.05 MB DOC)

Table S3 Strawberry varieties, soil sampling dates, soil types, and soil classification for field pairs in the study.
(0.05 MB DOC)

Table S4 Fruit, leaf, and sensory properties (mean ± standard error) for ‘Diamante’ and ‘San Juan’ strawberry cultivars from organic (ORG) and conventional (CON) farms in June and September 2004 and April, June, and September 2005.
(0.32 MB DOC)

Table S5 Soil properties (mean ± standard error) at two depths (0–10 cm and 20–30 cm) from organic and conventional strawberry farms in June 2004 and June 2005.
(0.09 MB DOC)

Data S1 Raw data for all slides.
(2.98 MB CSV)

Data S2 Data by functional groups (SNR>2.0).
(1.14 MB CSV)

Data S3 Normalized data by functional groups.
(0.00 MB CSV)

Data S4 Normalized data for diversity analysis.
(0.00 MB CSV)
