# Correction: Fruit and Soil Quality of Organic and Conventional Strawberry Agroecosystems

**DOI:** 10.1371/annotation/1eefd0a4-77af-4f48-98c3-2c5696ca9e7a

**Published:** 2010-10-06

**Authors:** John P. Reganold, Preston K. Andrews, Jennifer R. Reeve, Lynne Carpenter-Boggs, Christopher W. Schadt, J. Richard Alldredge, Carolyn F. Ross, Neal M. Davies, Jizhong Zhou

The leaf and fruit data in Table 1 were reported on a fresh-weight basis when they were actually on a dry-weight basis, due to a miscommunication with the lab that ran the analyses. These data are now correctly shown on a dry-weight basis. In addition, the dry-weight mineral data for strawberry fruit have been converted to fresh-weight data, statistically analyzed, and added to Table 1; please view the corrected Table 1 here: 

**Figure:**
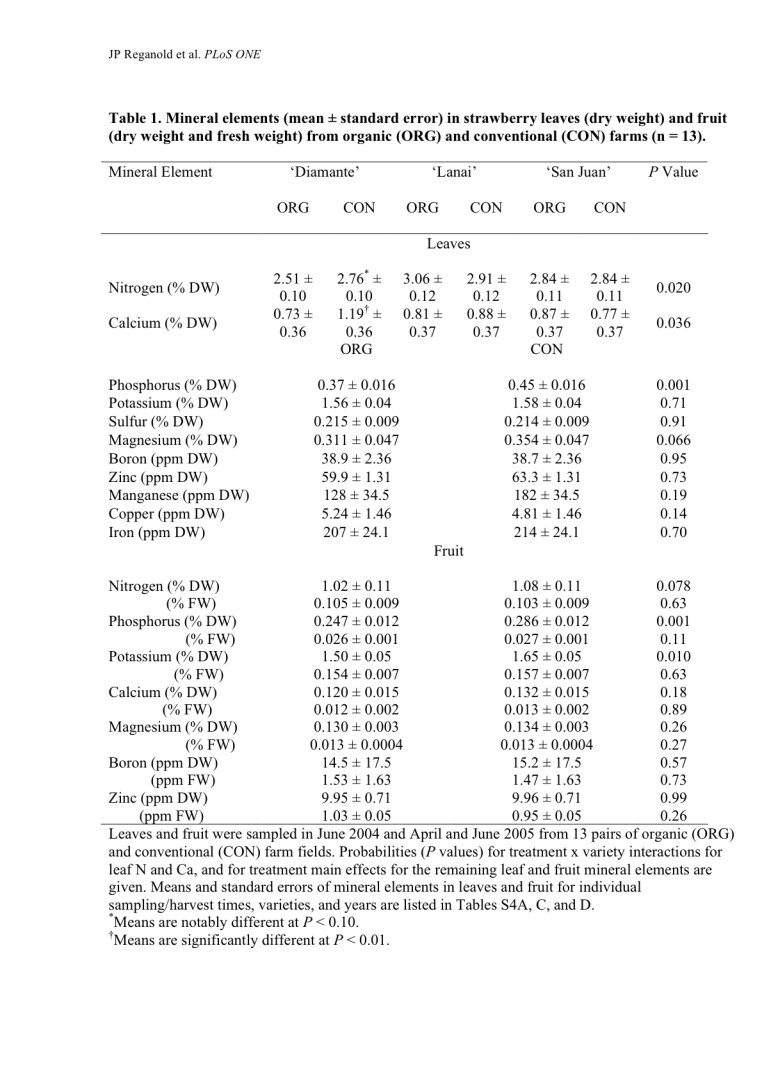


[^] Table S4 has also been revised to show the new fresh-weight fruit data for minerals; please view the corrected Table S4 here: 

[^] The new fresh-weight strawberry fruit data in Table 1 show no differences in phosphorus and potassium (or any other minerals) between organic and conventional systems, and therefore the statements about phosphorus and potassium in the Abstract and Results and Discussion sections have changed. 

