# Supplementary material for: Fruit and Soil Quality of Organic and Conventional Strawberry Agroecosystems
Source: PLoS One. 2010 Sep 1;5(9):e12346. doi: 10.1371/journal.pone.0012346 (PMC2931688; doi:10.1371/journal.pone.0012346)
Supplement: Table S1 — Two gene sequences endemic to conventionally managed field soils and 233 sequences endemic to organically managed field soils, and the organisms from which probes were designed. (0.15 MB DOC) [file pone.0012346.s001.doc]

**Table S1. Two gene sequences endemic to conventionally managed field soils and 233 sequences endemic to organically managed field soils, and the organisms from which probes were designed.**

| Organism | Gene |
| --- | --- |
| Endemic to Conventionally Farmed Soils | |
| Deinococcus radiodurans R1 | urease |
| Desulfitobacterium hafniense | tellurium |
| Endemic to Organically Farmed Soils | |
| Achromobacter cycloclastes | nosD |
| Acidiphilium multivorum | arsenic |
| Acinetobacter calcoaceticus | phenol-aerobic |
| Acinetobacter calcoaceticus | toluene-aerobic |
| Agrobacterium tumefaciens (Rhizobium radiobacter) | protocatechuate |
| Agrobacterium tumefaciens str. C58 | copper |
| Agrobacterium tumefaciens str. C58 | urease |
| Alcaligenes faecalis | phenanthrene |
| Alicyclobacillus acidocaldarius | cellulase |
| Anaeromyxobacter dehalogenans 2CP-C | cytochrome |
| Archaeoglobus fulgidus DSM 4304 | atrazine |
| Arthrobacter keyseri | phthalate |
| Azospirillum brasilense | norB |
| Azotobacter vinelandii | arsenic |
| Azotobacter vinelandii | benzoate-anaerobic |
| Azotobacter vinelandii | phenylpropionate |
| Azotobacter vinelandii | tellurium |
| Bacillus cereus ATCC 14579 | rbcL |
| Bacillus circulans | chitinase |
| Bacillus halodurans C-125 | aluminum |
| Bacillus sp. | cellulase |
| Bacillus sp. | urease |
| Bacillus sp. TB-90 | urease |
| Bacillus subtilis | chitinase |
| Bacillus subtilis subsp. subtilis str. 168 | arsenic |
| Bacillus subtilis subsp. subtilis str. 168 | tellurium |
| Bdellovibrio bacteriovorus HD100 | methyl tert-butyl ether |
| Bordetella bronchiseptica RB50 | benzoate-anaerobic |
| Bordetella bronchiseptica RB50 | naphthalene |
| Bordetella parapertussis | urease |
| Bordetella parapertussis 12822 | benzoate-anaerobic |
| Bradyrhizobium japonicum USDA 110 | homogentisate |
| Bradyrhizobium japonicum USDA 110 | protocatechuate |
| Bradyrhizobium japonicum USDA 110 | thiocyanate |
| Brevibacterium sp. HCU | cyclohexanol |
| Brucella melitensis 16M | urease |
| Burkholderia cepacia | nitrotoluene |
| Burkholderia cepacia | phthalate |
| Burkholderia kururiensis | phenol-aerobic |
| Burkholderia sp. BZN2 | benzene |
| Burkholderia sp. RASC | nitrotoluene |
| Burkholderia sp. RP007 | phenanthrene |
| Caldibacillus cellulovorans | mannanase |
| Candida albicans | chitinase |
| Chlamydophila pneumoniae TW-183 | pentachlorophenol |
| Clostridium cellulolyticum | cellulase |
| Clostridium thermocellum ATCC 27405 | cellulase |
| Cordyceps bassiana (anamorph: Beauveria bassiana) | chitinase |
| Corynebacterium efficiens YS-314 | urease |
| Corynebacterium efficiens YS-314 | catechol-ortho derivative |
| Cycloclasticus sp. A5 | phenanthrene |
| Daedalea quercina | laccase |
| Desulfitobacterium dehalogenans | chlorophenol |
| Desulfitobacterium hafniense | cellulase |
| Desulfitobacterium hafniense DCB-2 | cytochrome |
| Desulfitobacterium sp. PCE1 | tri- or tetrachloroethylene |
| Desulfovibrio desulfuricans | dsrA |
| Desulfovibrio desulfuricans G20 | cytochrome |
| Desulfovibrio fructosovorans | dsrA |
| Enterococcus faecalis V583 | FTHFS |
| Epidinium caudatum | cellulase |
| Escherichia coli | urease |
| Escherichia coli O157:H7 EDL933 | urease |
| Geobacter metallireducens | cytochrome |
| Geobacter metallireducens GS-15 | cytochrome |
| Geobacter sulfurreducens PCA | cytochrome |
| Gloeobacter violaceus PCC 7421 | catechol-ortho derivative |
| Haloarcula marismortui | urease |
| Hypocrea jecorina | cellulase |
| Hypocrea jecorina (anamorph: Trichoderma reesei) | cellulase |
| IncN plasmid R46 | arsenic |
| Klebsiella pneumoniae | silver |
| Lab clone | dsrB |
| Lab clone | nirK |
| Lactobacillus plantarum WCFS1 | copper |
| Lactobacillus plantarum WCFS1 | narG |
| Lactococcus lactis subsp. lactis Il1403 | benzoate-anaerobic |
| Legionella pneumophila | copper |
| Leptospira interrogans serovar Lai str. 56601 | benzoate-anaerobic |
| Leuconostoc mesenteroides subsp. mesenteroides ATCC 8293 | arsenic |
| Leuconostoc mesenteroides subsp. mesenteroides ATCC 8293 | cellulase |
| Leuconostoc mesenteroides subsp. mesenteroides ATCC 8293 | chromium |
| Leuconostoc mesenteroides subsp. mesenteroides ATCC 8293 | cyanuric acid |
| Leuconostoc mesenteroides subsp. mesenteroides ATCC 8293 | tellurium |
| Magnetospirillum magnetotacticum | dodecyl sulfate |
| Magnetospirillum magnetotacticum | tellurium |
| Magnetospirillum magnetotacticum | urease |
| Metagenome clone | unknown |
| Metallosphaera sedula | unknown |
| Methanocaldococcus jannaschii DSM 2661 | rbcL |
| Methanococcus vannielii | mcrA |
| Methanopyrus kandleri AV19 | nifH |
| Methanopyrus kandleri AV19 | mcr |
| Methanosarcina acetivorans C2A | nickel |
| Methanosarcina barkeri | unknown |
| Methanosarcina mazei Go1 | CO-dehydrogenase |
| Methanosarcina mazei Go1 | thiocyanate |
| Methanothermobacter thermautotrophicus | mcrA |
| Methanothermobacter thermautotrophicus str. Delta H | thiocyanate |
| Methylomonas sp. KSWIII | mmo |
| Mycobacterium bovis AF2122/97 | aniline |
| Mycobacterium leprae | 4-chlorobenzoate |
| Mycobacterium leprae TN | catechol-ortho derivative |
| Mycobacterium leprae TN | Pgl |
| Mycobacterium tuberculosis H37Rv | dichloroethane |
| Mycobacterium tuberculosis H37Rv | mercury |
| Mycobacterium vanbaalenii | phthalate |
| Neisseria meningitidis MC58 | FTHFS |
| Neisseria meningitidis Z2491 | arsenic |
| Neurospora crassa | phenol-aerobic |
| Neurospora crassa | pentachlorophenol |
| Nitrosococcus halophilus | amoA |
| Nitrosococcus sp. C-113 | amoA |
| Nitrosomonas sp. Nm143 | amoA |
| Nocardioides sp. KP7 | phenanthrene |
| Nostoc punctiforme | tellurium |
| Nostoc sp. PCC 7120 | mercury |
| Novosphingobium aromaticivorans | cresol |
| Paenibacillus ehimensis | chitinase |
| Paracoccus denitrificans | glyphosate |
| Pelotomaculum sp. MGP | dsrA |
| Pelotomaculum sp. MGP | dsrB |
| Penicillium griseoroseum | polygalacturonase |
| Penicillium janthinellum | cellulase |
| Photorhabdus luminescens subsp. laumondii TTO1 | phenylpropionate |
| Photorhabdus luminescens subsp. laumondii TTO1 | thiocyanate |
| Phytophthora cinnamomi | polygalacturonase |
| Piromyces sp. E2 | cellulase |
| Plasmid RK2Te-r | tellurium |
| Prauserella rugosa | methyl tert-butyl ether |
| Prochlorococcus marinus str. MIT 9313 | mcrA |
| Prochlorococcus marinus str. MIT 9313 | urease |
| Prochlorococcus marinus subsp. pastoris str. CCMP1986 | urease |
| Proteus mirabilis | tellurium |
| Proteus vulgaris | phenol-aerobic |
| Pseudoalteromonas sp. S9 | chitinase |
| Pseudomonas aeruginosa | benzoate-anaerobic |
| Pseudomonas aeruginosa | biphenyl |
| Pseudomonas aeruginosa UCBPP-PA14 | chromium |
| Pseudomonas chlororaphis | cymene |
| Pseudomonas fluorescens | arsenic |
| Pseudomonas fluorescens | cellulase |
| Pseudomonas fluorescens | methyl tert-butyl ether |
| Pseudomonas pseudoalcaligenes | biphenyl |
| Pseudomonas putida | aniline |
| Pseudomonas putida | haloacid |
| Pseudomonas putida | isopropylbenzene |
| Pseudomonas putida | nitrobenzene |
| Pseudomonas putida | nitrobenzoate |
| Pseudomonas putida | octane |
| Pseudomonas putida | toluene-aerobic |
| Pseudomonas putida | toluene-aerobic |
| Pseudomonas putida KT2440 | nickel |
| Pseudomonas sp. CBS3 | haloacid |
| Pseudomonas sp. KL28 | catechol-meta derivative |
| Pseudomonas sp. TW3 | nitrotoluene |
| Pseudomonas stutzeri | phenol-aerobic |
| Pyrococcus furiosus | cellulase |
| Ralstonia sp. E2 | phenol-aerobic |
| Rhizobium sp. | haloacid |
| Rhodobacter sphaeroides | chromium |
| Rhodococcus erythropolis | cadmium |
| Rhodococcus erythropolis | limonene |
| Rhodococcus opacus | catechol-ortho derivative |
| Rhodococcus rhodochrous | benzonitrile |
| Rhodococcus sp. 19070 | benzoate-aerobic |
| Rhodococcus sp. N-771 | benzonitrile |
| Rhodococcus sp. RHA1 | biphenyl |
| Rhodococcus sp. RHA1 | catechol-meta derivative |
| Rhodopseudomonas palustris CGA009 | benzonitrile |
| Rhodopseudomonas palustris CGA009 | urease |
| Roseovarius nubinhibens | vanillin |
| Salmonella typhimurium LT2 | dimethyl sulfoxide |
| Schizosaccharomyces pombe (fission yeast) | urease |
| Schizosaccharomyces pombe (fission yeast) | zinc, cadmium |
| Sinorhizobium meliloti (Rhizobium meliloti) | rbcS |
| Sphingomonas chungbukensis | cresol |
| Sphingomonas paucimobilis | dibenzofuran |
| Sphingomonas sp. CB3 | carbazole |
| Sphingomonas sp. Spi7 | mercury |
| Stigmatella aurantiaca | acetylene |
| Streptococcus agalactiae 2603V/R | arsenic |
| Streptococcus pyogenes M1 GAS | benzoate-anaerobic |
| Streptomyces atroolivaceus | cyanamide |
| Streptomyces coelicolor A3(2) | 3-chlorobenzoate |
| Streptomyces coelicolor A3(2) | atrazine |
| Streptomyces coelicolor A3(2) | benzoate-anaerobic |
| Streptomyces reticuli | cellulase |
| Streptomyces setonii | catechol |
| Streptomyces sp. NRRL 5331 | mercury |
| Sulfolobus solfataricus P2 | copper |
| Sulfolobus solfataricus P2 | haloacid |
| Sulfolobus solfataricus P2 | toluene-aerobic |
| Terrabacter sp. YK3 | dibenzofuran |
| Thauera aromatica | toluene-anaerobic |
| Thermobifida fusca | catechol-ortho derivative |
| Thermobifida fusca | cellulase |
| Thermosynechococcus elongatus BP-1 | arsenic |
| Thermosynechococcus elongatus BP-1 | narB |
| Thermosynechococcus elongatus BP-1 | urease |
| Thermotoga maritima MSB8 | cellulase |
| Tropheryma whipplei str. Twist | benzoate-anaerobic |
| Uncultured bacterium | dsrA |
| Uncultured bacterium | FTHFS |
| Uncultured bacterium | haloacid |
| Uncultured bacterium | narG |
| Uncultured bacterium | nasA |
| Uncultured bacterium | nifH |
| Uncultured bacterium | nirK |
| Uncultured bacterium | nirS |
| Uncultured bacterium | norB |
| Uncultured basidiomycete | laccase |
| Uncultured deep-sea autotrophic bacterium TAGI-2 | rbcL |
| Uncultured methanogenic archaeon | mcrA |
| Uncultured organism | acrylonitrile |
| Uncultured organism | nirK |
| Uncultured soil bacterium | nosZ |
| Uncultured sulfate-reducing bacterium | dsrA |
| Uncultured sulfate-reducing bacterium | dsrB |
| Uncultured temperate forest soil bacterium CZ1441 | nosZ |
| Unidentified bacterium | nifH |
| Unidentified nitrogen-fixing bacteria | nifH |
| Vibrio cholerae | homogentisate-derivative |
| Wautersia eutropha | 2,4-D |
| Xanthomonas axonopodis pv. citri str. 306 | arsenic |
| Xanthomonas campestris | copper |
| Xanthomonas campestris pv. campestris str. ATCC 33913 | cobalt, zinc, cadmium |
| Xanthomonas campestris pv. campestris str. ATCC 33913 | copper |
| Xylella fastidiosa 9a5c | cobalt, zinc, cadmium |
| Xylella fastidiosa Dixon | copper |
| Xylella fastidiosa Temecula1 | benzene |
| Yersinia pestis CO92 | aniline |
| Yersinia pestis KIM | tellurium |

Metals indicate genes involved with reduction of or organismal resistance to the metal. Chemical compounds indicate genes involved with degradation of the compound.
