# Supplementary material for: Fruit and Soil Quality of Organic and Conventional Strawberry Agroecosystems
Source: PLoS One. 2010 Sep 1;5(9):e12346. doi: 10.1371/journal.pone.0012346 (PMC2931688; doi:10.1371/journal.pone.0012346)
Supplement: Table S2 — Agrichemical inputs (insecticides, fungicides, herbicides, molluscides, adjuvants, fumigants, and fertilizers) applied to 26 strawberry fields during the 2004 and 2005 growing seasons. (0.05 MB DOC) [file pone.0012346.s002.doc]

**Table S2. Agrichemical inputs (insecticides, fungicides, herbicides, molluscides, adjuvants, fumigants, and fertilizers) applied to 26 strawberry fields during the 2004 and 2005 growing seasons.**

| Agrichemical category | Organic^*^ (13 total fields) | | Conventional (13 total fields) | |
| --- | --- | --- | --- | --- |
|  | 2004 (5 fields) | 2005 (8 fields) | 2004 (5 fields) | 2005 (8 fields) |
| Insecticides/miticides | Azadirachtin (2), *Bacillus thuringiensis aizawai* (3), cottonseed-clove-garlic oils (2), fatty acids+K-salts (1), neem oil (2), pyrethrins (3), rotenone (1), spinosad (2) | Azadirachtin (4), *Beauveria bassiana* (1), *Bacillus thuringiensis aizawai* (1), *B. thuringiensis kurstaki* (1), neem oil (3), pyrethrins (4), rotenone (1), spinosad (5 | 1, 3-dichloropropene, abamectin (1), avermectine (2), *Bacillus thuringiensis aizawai* (2), bifenazate (2), bifenthrin (3), fenpropathrin (3), hexythiazox (1), malathion (5), methomyl (4), naled (3), pyriproxyfen (1), spinosad (2), xylene (1) | Avermectine (5), azadirachtin (1), *Bacillus thuringiensis aizawai* (6), *B. thuringiensis kurstaki* (6), bifenazate (6), bifenthrin (3), diazinon (2), extoxazole (4), fenpropathrin (6), hexythiazox (2), imidacloprid (1), malathion (8), methomyl (5), naled (1), pyriproxyfen (1), spinosad (8), spiromesifen (4), sulfur (1), xylene (1) |
| Fungicides | Neem oil (2), potassium bicarbonate (5), sulfur (5) | *Bacillus pumilis* (4), *B. subtilis* (1), neem oil (3), potassium bicarbonate (5), rosemary oil (1), sulfur (8) | Azoxystrobin (1), boscalid (3), captan (5), cyprodinil (1), dimethyl 4,4'-o-phenylenebis[3-thioallophanate] (1), fenhexamid (4), fludioxonil (1), fosetyl-Al (3), harpin protein (1), mefenoxam (2), myclobutanil (4), pyraclostrobin (4), sulfur (4), thiram (3), triflumazole (1) | Azoxystrobin (3), *Bacillus pumilis* (3), *B. subtilis* (3), boscalid (5), captan (8), cyprodinil (1), dimethyl 4,4'-o-phenylenebis[3-thioallophanate] (2), fenhexamid (8), fludioxonil (1), fosetyl-Al (3), mefenoxam (3), myclobutanil (4), potassium bicarbonate (2), pyraclostrobin (5), sulfur (8), thiophanate-methyl (3), thiram (3), triflumazole (2) |
| Herbicides | None applied | None applied | Paraquat dichloride (1) | Napropamide (2) |
| Molluscide | None applied | None applied | Metaldehyde (1) | Metaldehyde (3) |
| Adjuvants | Poly-1-p-menthene (2) | Gypsum (1), poly-1-p-menthene (2) | Dimethylpolysiloxane (1), octyl-phenoxy-polyethoxy ethanol, isopropanol, linear alkyl sulfonate, buffering acids, coconut oil amine condensate (1) | Octyl-phenoxy-polyethoxy ethanol, isopropanol, linear alkyl sulfonate, buffering acids, coconut oil amine condensate (5), phosphatidylcholine, methyl acetic acid, alkyl polyoxyethylene ether (1), polyether-polymethyl siloxane copolymer (1), poly-1-p-menthene (2) |
| Fumigants | None applied | None applied | All 5 farm fields used methyl bromide, with 4 using methyl bromide with chloropicrin and 1 using methyl bromide alone. | All 8 farm fields used methyl bromide, with 5 using methyl bromide with chloropicrin and 3 using methyl bromide alone. |
| Fertilizers | 20.2-24.6 Mg compost^†^ ha^-1^ (5), gypsum (2), humic acid (1), kelp extract (2), liquid fish emulsion (2), liquid fertilizer derived from bloodmeal, and feathermeal (5), greensand (2), sulfate of potash (1) | 20.2-24.6 Mg compost^†^ ha^-1^ (8), boron (1), gypsum (2), humic acid (1), kelp extract (2), liquid fish emulsion (5), liquid fertilizer derived from bloodmeal, and feathermeal (6), greensand (2), sulfate of potash (1) | 11.2-13.4 Mg compost^†^ ha^-1^ (5), ammonium sulfate (2), calcium (1), calcium thiosulfate (2), kelp extract (1), N-P-K (3), nitrogen (5), potassium (2), potassium nitrate (1), sulfur (2), urea (2) | 11.2-13.4 Mg compost^†^ ha^-1^ (8), ammonium sulfate (3), *Ascophyllum nodosum* extract (6), boron (1), calcium (5), calcium ammonium sulfate (3), calcium thiosulfate (1), humic acid (4), kelp extract (4), N-P-K (8), nitrogen (8), potassium (3), sodium borate (1), urea (3) |

Numbers in parentheses are the number of farm fields applying that agrichemical at least once during the growing season.

^*^All organic farms and agrichemicals were certified organic under the USDA National Organic Program guidelines.

^†^Compost for the organic and conventional farms was similar and mostly consisted of a green waste (yard and park trimmings), such as [grass](http://en.wikipedia.org/wiki/Grass), [flower](http://en.wikipedia.org/wiki/Flower) cuttings, and [hedge](http://en.wikipedia.org/wiki/Hedge_(barrier)) trimmings, purchased from local, commercial composting facilities.
