# Supplementary material for: Fruit and Soil Quality of Organic and Conventional Strawberry Agroecosystems
Source: PLoS One. 2010 Sep 1;5(9):e12346. doi: 10.1371/journal.pone.0012346 (PMC2931688; doi:10.1371/journal.pone.0012346)
Supplement: Table S3 — Strawberry varieties, soil sampling dates, soil types, and soil classification for field pairs in the study. (0.05 MB DOC) [file pone.0012346.s003.doc]

**Table S3. Strawberry varieties, soil sampling dates, soil types, and soil classification for field pairs in the study.**

| Field Pair | Strawberry Variety | | Soil Sampling Dates | Soil Type | Soil Classification |
| --- | --- | --- | --- | --- | --- |
| 1 | Diamante | June 2004 | | Baywood loamy sand | Sandy, mixed, thermic Entic Haploxerolls |
| 2 | Diamante | June 2004 | | Baywood loamy sand | Sandy, mixed, thermic Entic Haploxerolls |
| 3 | San Juan | June 2004 | | Salinas silty clay loam | Fine-loamy, mixed, superactive, thermic Pachic Haploxerolls |
| 4 | San Juan | June 2004 | | Baywood loamy sand | Sandy, mixed, thermic Entic Haploxerolls |
| 5 | Diamante | June 2004 | | Baywood loamy sand | Sandy, mixed, thermic Entic Haploxerolls |
| 6 | Diamante | April, June 2005 | | Baywood loamy sand | Sandy, mixed, thermic Entic Haploxerolls |
| 7 | Diamante | April, June 2005 | | Baywood loamy sand | Sandy, mixed, thermic Entic Haploxerolls |
| 8 | Diamante | April, June 2005 | | Baywood loamy sand | Sandy, mixed, thermic Entic Haploxerolls |
| 9 | Lanai | April, June 2005 | | Elder sandy loam | Coarse-loamy, mixed, superactive, thermic Cumulic Haploxerolls |
| 10 | Lanai | April, June 2005 | | Salinas silty clay loam | Fine-loamy, mixed, superactive, thermic Pachic Haploxerolls |
| 11 | Lanai | April, June 2005 | | Salinas silty clay loam | Fine-loamy, mixed, superactive, thermic Pachic Haploxerolls |
| 12 | San Juan | April, June 2005 | | Baywood loamy sand | Sandy, mixed, thermic Entic Haploxerolls |
| 13 | San Juan | April, June 2005 | | Salinas silty clay loam | Fine-loamy, mixed, superactive, thermic Pachic Haploxerolls |

Soil types and classification were obtained from field analyses and U.S. government soil survey reports [1,2].

1. Cook TD, Beutler CS (1978) *Soil Survey of Monterey County, California.* Washington DC: USDA Soil Conservation Service.

2. Bowman RH, Estrada DC (1980) *Soil Survey of Santa Cruz County, California.* Washington DC: USDA Soil Conservation Service.
