# Supplementary material for: Fruit and Soil Quality of Organic and Conventional Strawberry Agroecosystems
Source: PLoS One. 2010 Sep 1;5(9):e12346. doi: 10.1371/journal.pone.0012346 (PMC2931688; doi:10.1371/journal.pone.0012346)
Supplement: Table S4 — Fruit, leaf, and sensory properties (mean ± standard error) for ‘Diamante’ and ‘San Juan’ strawberry cultivars from organic (ORG) and conventional (CON) farms in June and September 2004 and April, June, and September 2005. (0.32 MB DOC) [file pone.0012346.s004.doc]

**Table S4A. Fruit and leaf properties (mean ± standard error) for ‘Diamante’ and ‘San Juan’ strawberry cultivars from organic (ORG) and conventional (CON) farms** in June 2004.

| Fruit and Leaf Properties (units) | ‘Diamante’ (n=3) | | ‘San Juan’ (n=2) | |
| --- | --- | --- | --- | --- |
|  | ORG | CON | ORG | CON |
| Fruit fresh weight (g) | 27.6±0.58 | 29.6±1.66 | 26.9±4.80 | 29.1±1.66 |
| Dry matter (%) | 10.3±0.003 | 9.12±0.004 | 9.91±0.016 | 9.28±0.004 |
| Fruit weight loss (%) | 26.3±0.68 | 33.6±1.23 | 28.4±3.63 | 22.5±0.85 |
| Fruit firmness (N) | 3.02±0.19 | 2.64±0.16 | 2.98±0.38 | 2.92±0.04 |
| External L* (+60 to -60) | 35.7±0.74 | 39.5±0.43 | 38.3±1.45 | 37.0±1.32 |
| External C* (+60 to -60) | 44.9±0.99 | 45.4±1.21 | 39.1±1.35 | 40.6±1.32 |
| External h_ab_ (^o^) | 29.8±0.60 | 32.9±0.38 | 34.9±0.38 | 32.9±0.79 |
| Internal L* (+60 to -60) | 66.7±0.48 | 66.3±0.30 | 67.2±0.57 | 68.5±0.05 |
| Internal C* (+60 to -60) | 17.1±0.60 | 16.9±0.25 | 23.0±0.09 | 21.5±0.11 |
| Internal h_ab_ (^o^) | 56.7±1.92 | 61.5±1.44 | 61.8±1.82 | 62.5±1.61 |
| Soluble solids (^o^brix) | 8.14±0.58 | 8.04±0.18 | 9.96±1.29 | 7.62±0.84 |
| TA (mg citric acid g^-1^ FW) | 9.31±0.30 | 8.35±0.40 | 7.29±0.60 | 7.43±0.13 |
| Soluble solids/TA | 1.16±0.14 | 0.93±0.03 | 0.88±0.06 | 1.09±0.06 |
| pH | 3.69±0.02 | 3.68±0.01 | 3.93±0.002 | 3.95±0.02 |
| Reducing sugars (mg Glc g^-1^ FW) | 66.6±7.47 | 64.8±0.91 | 73.0±5.32 | 60.8±1.09 |
| Total sugars (mg Glc g^-1^ FW) | 68.4±2.28 | 67.1±5.79 | 79.3±6.84 | 69.5±1.09 |
| Total antioxidant activity  (mmol Trolox equiv/g FW)  Hydrophilic  Lipophilic | 13.6±1.30  10.9±1.00  2.72±0.36 | 11.3±0.90  9.16±0.74  2.10±0.22 | 11.5±0.79  9.49±0.52  1.99±0.27 | 10.5±0.27  8.75±0.38  1.71±0.10 |
| Total phenolics  (mg gallic acid equiv/g FW) | 1.74±0.03 | 1.46±0.09 | 1.43±0.10 | 1.36±0.05 |
| Total anthocyanins  (μg P-3-Glc^*^ equivalents/g FW) | 171±7.2 | 137±4.7 | 191±2.1 | 201±40.1 |
| Total ascorbic acid (mg/g FW)  Reduced AsA  Oxidized dehydro-AsA  Reduced AsA/total AsA | 0.53±0.05  0.36±0.01  0.17±0.06  0.69±0.08 | 0.45±0.01  0.36±0.03  0.09±0.01  0.79±0.04 | 0.60±0.05  0.39±0.06  0.20±0.01  0.65±0.04 | 0.61±0.01  0.44±0.02  0.17±0.01  0.73±0.02 |
| Ellagic acid (mg 100 g^-1^ FW)  Glycoside  Total | 3.10±0.99  36.4±2.38  39.5±1.39 | 2.17±0.95  52.6±13.1  54.8±13.3 | 6.75±4.62  27.9±17.9  34.7±13.3 | 2.00±0.71  66.9±15.2  68.9±15.9 |
| Quercetin (mg 100 g^-1^ FW)  Glycoside  Total | 2.64±0.07  4.76±0.41  7.40±0.38 | 2.46±0.02  5.45±0.22  7.91±0.24 | 2.89±0.06  4.51±1.24  7.40±1.30 | 2.29±0.18  4.74±0.68  7.03±0.50 |
| Kaempferol (mg 100 g^-1^ FW)  Glycoside  Total | 1.08±0.05  3.10±1.17  4.18±1.16 | 0.95±0.05  3.17±1.08  4.12±1.12 | 0.93±0.12  5.34±0.08  6.27±0.20 | 0.86±0.05  4.40±0.12  5.26±0.17 |
| Ploretin (mg 100 g^-1^ FW)  Glycoside (phloridzin)  Total | 2.33±0.03  1.27±0.08  3.60±0.11 | 2.27±0.05  2.05±0.32  4.32±0.28 | 2.27±0.10  1.57±0.08  3.84±0.02 | 2.14±0.12  1.40±0.32  3.54±0.19 |
| Naringenin (mg 100 g^-1^ FW)  R-enantomer  S-enantomer  Glycoside (naringin)  R-enantomer  S-enantomer  Total  R-enantomer  S-enantomer | 0.63±0.22  0.48±0.22  0.69±0.19  0.67±0.20  1.32±0.04  1.15±0.03 | 0.42±0.08  0.28±0.10  1.12±0.02  1.04±0.11  1.54±0.06  1.32±0.02 | 0.51±0.14  0.40±0.20  0.42±0.28  0.61±0.25  0.93±0.41  1.00±0.05 | 0.29±0.04  0.15±0.05  1.69±0.56  1.75±0.60  1.98±0.60  1.90±0.65 |
| Fruit nitrogen (% FW) | 0.84±0.09 | 0.83±0.05 | 1.06±0.03 | 1.07±0.13 |
| Fruit phosphorus (% FW) | 0.21±0.02 | 0.23±0.01 | 0.26±0.02 | 0.27±0.02 |
| Fruit potassium (% FW) | 1.51±0.12 | 2.25±0.23 | 1.57±0.03 | 1.68±0.06 |
| Fruit calcium (% FW) | 0.12±0.003 | 0.16±0.02 | 0.15±0.02 | 0.13±0.02 |
| Fruit magnesium (% FW) | 0.13±0.003 | 0.12±0.01 | 0.14±0.00 | 0.14±0.01 |
| Fruit boron (ppm) | 10.3±0.88 | 11.3±0.33 | 25.0±6.00 | 16.5±0.50 |
| Fruit zinc (ppm) | 7.00±0.58 | 8.00±0.58 | 11.5±0.50 | 11.5±0.50 |
| Leaf nitrogen (% FW) | 2.38±0.05 | 2.45±0.19 | 2.62±0.02 | 2.59±0.14 |
| Leaf phosphorus (% FW) | 0.31±0.02 | 0.35±0.01 | 0.38±0.05 | 0.34±0.03 |
| Leaf potassium (% FW) | 1.48±0.04 | 1.67±0.17 | 1.40±0.07 | 1.37±0.13 |
| Leaf calcium (% FW) | 0.72±0.03 | 0.89±0.04 | 0.75±0.04 | 0.88±0.23 |
| Leaf magnesium (% FW) | 0.29±0.01 | 0.31±0.02 | 0.33±0.02 | 0.34±0.10 |
| Leaf sulfur (% FW) | 0.20±0.05 | 0.17±0.02 | 0.19±0.00 | 0.18±0.01 |
| Leaf boron (ppm) | 27.3±4.48 | 28.0±1.00 | 62.0±14.0 | 49.0±25.0 |
| Leaf zinc (ppm) | 12.7±1.20 | 12.7±1.20 | 21.5±2.50 | 15.0±4.00 |
| Leaf manganese (ppm) | 122±11.3 | 225±57.3 | 100±3.50 | 164±87.0 |
| Leaf copper (ppm) | 5.00±0.58 | 4.33±0.33 | 7.00±1.00 | 4.50±0.50 |
| Leaf iron (ppm) | 181±44.9 | 168±27.5 | 156±42.0 | 199±36.0 |

**Table S4B. Fruit and sensory properties (mean ± standard error) for ‘Diamante’ and ‘San Juan’ strawberry cultivars from organic (ORG) and conventional (CON) farms** in September 2004.

| Fruit and Sensory Properties (units) | ‘Diamante’ (n=3) | | ‘San Juan’ (n=2) | |
| --- | --- | --- | --- | --- |
|  | ORG | CON | ORG | CON |
| Fruit fresh weight (g) | 21.9±0.83 | 26.3±1.15 | 19.5±0.26 | 18.1±2.98 |
| Dry matter (%) | 9.31±0.007 | 8.12±0.001 | 9.64±0.001 | 9.11±0.007 |
| Fruit weight loss (%) | 38.1±2.93 | 40.0±3.21 | 28.2±4.75 | 38.5±2.43 |
| Fruit firmness (N) | 2.62±0.17 | 2.26±0.19 | 2.51±0.20 | 2.71±0.008 |
| External L* (+60 to -60) | 40.2±1.86 | 40.6±1.52 | 38.9±0.40 | 39.5±1.91 |
| External C* (+60 to -60) | 44.2±0.40 | 43.1±1.57 | 38.9±0.10 | 39.5±1.86 |
| External h_ab_ (^o^) | 33.7±1.34 | 34.1±0.85 | 32.9±0.19 | 33.1±1.08 |
| Internal L* (+60 to -60) | 67.8±0.95 | 64.7±0.76 | 67.0±0.35 | 63.9±0.58 |
| Internal C* (+60 to -60) | 17.9±1.86 | 19.5±1.05 | 25.4±1.04 | 28.8±2.11 |
| Internal h_ab_ (^o^) | 59.6±4.35 | 61.5±1.91 | 54.2±2.40 | 51.3±2.64 |
| Soluble solids (^o^brix) | 8.14±0.54 | 7.51±0.12 | 8.12±0.63 | 9.17±1.02 |
| TA (mg citric acid g^-1^ FW) | 8.28±0.46 | 7.06±0.37 | 7.04±0.07 | 8.56±0.11 |
| Soluble solids/TA | 1.07±0.13 | 1.04±0.04 | 1.10±0.14 | 1.11±0.07 |
| pH | 3.73±0.01 | 3.78±0.03 | 3.92±0.06 | 3.89±0.03 |
| Reducing sugars (mg Glc g^-1^ FW) | 59.8±2.91 | 62.1±5.71 | 73.1±3.57 | 57.9±3.29 |
| Total sugars (mg Glc g^-1^ FW) | 59.6±3.15 | 65.8±1.06 | 70.2±0.36 | 59.2±2.13 |
| Total antioxidant activity (mmol Trolox equiv/g FW)  Hydrophilic  Lipophilic | 11.1±0.27  8.88±0.26  2.22±0.05 | 11.1±0.55  8.94±0.47  2.20±0.09 | 10.3±0.57  8.79±0.47  1.54±0.10 | 10.8±0.35  8.90±0.16  1.88±0.19 |
| Total phenolics  (mg gallic acid equiv/g FW) | 1.39±0.06 | 1.22±0.05 | 1.32±0.19 | 1.29±0.07 |
| Total anthocyanins  (μg P-3-Glc^*^ equivalents/g FW) | 185±11.7 | 146±4.0 | 262±10.8 | 255±0.4 |
| Total ascorbic acid (mg/g FW)  Reduced AsA  Oxidized dehydro-AsA  Reduced AsA/total AsA | 0.54±0.04  0.34±0.02  0.20±0.03  0.64±0.02 | 0.39±0.04  0.29±0.02  0.10±0.03  0.75±0.04 | 0.59±0.03  0.44±0.0040.14±0.02  0.76±0.03 | 0.57±0.11  0.43±0.06  0.14±0.04  0.76±0.03 |
| Ellagic acid (mg 100 g^-1^ FW)  Glycoside  Total | 3.96±1.24  33.2±8.63  37.2±8.07 | 1.29±0.59  40.9±13.3  42.2±12.7 | 2.54±2.33  182±20.4  185±18.1 | 2.97±0.04  106±24.1  109±24.1 |
| Quercetin (mg 100 g^-1^ FW)  Glycoside  Total | 3.04±0.22  3.50±1.11  6.54±0.93 | 2.68±0.07  4.94±0.58  7.62±0.62 | 2.70±0.13  4.82±0.83  7.52±0.96 | 2.71±0.19  5.59±1.36  8.30±1.56 |
| Kaempferol (mg 100 g^-1^ FW)  Glycoside  Total | 1.02±0.12  3.30±1.31  4.33±1.14 | 0.90±0.02  4.52±0.22  5.42±0.23 | 0.86±0.02  1.08±0.32  1.94±0.34 | 0.98±0.01  1.37±0.66  2.35±0.64 |
| Ploretin (mg 100 g^-1^ FW)  Glycoside (phloridzin)  Total | 2.62±0.16  0.66±0.24  3.27±0.09 | 2.37±0.04  1.53±0.20  3.90±0.22 | 2.32±0.01  1.29±0.02  3.61±0.04 | 2.37±0.02  1.87±0.10  4.24±0.08 |
| Naringenin (mg 100 g^-1^ FW)  S-enantomer  R-enantomer  Glycoside (naringin)  S-enantomer  R-enantomer  Total  S-enantomer  R-enantomer | 0.64±0.30  0.42±0.30  1.28±0.39  1.10±0.52  1.92±0.69  1.52±0.81 | 0.88±0.45  0.68±0.48  0.88±0.42  0.69±0.30  1.76±0.14  1.37±0.21 | 0.41±0.01  0.14±0.001  6.03±4.93  6.16±5.16  6.44±4.91  6.30±5.17 | 0.34±0.04  0.14±0.02  1.13±0.22  0.94±0.17  1.47±0.26  1.08±0.19 |
| Overall acceptance^*^ | 5.87±0.51 | 4.73±0.48 | 5.94±0.36 | 6.37±0.12 |
| Flavor^*^ | 5.67±0.43 | 4.73±0.55 | 5.75±0.30 | 6.45±0.30 |
| Juiciness^*^ | 5.98±0.37 | 5.70±0.40 | 6.45±0.40 | 6.75±0.20 |
| Sweetness^*^ | 5.45±0.55 | 4.33±0.44 | 5.20±0.25 | 5.50±0.30 |
| Tartness^*^ | 4.78±0.32 | 4.73±0.29 | 4.10±0.20 | 4.95±0.15 |
| Appearance^*^ | 6.91±0.72 | 5.34±0.63 | 6.85±0.05 | 6.42±0.92 |

^*^Nine-point hedonic/intensity scale.

**Table S4C. Fruit, leaf, and sensory properties (mean ± standard error) for ‘Diamante’, ‘Lanai’, and ‘San Juan’ strawberry cultivars from organic (ORG) and conventional (CON) farms** in April 2005.

| Fruit, Leaf, and Sensory Properties (units) | ‘Diamante’ (n=3) | | ‘Lanai’ (n=3) | | ‘San Juan’ (n=2) | |
| --- | --- | --- | --- | --- | --- | --- |
|  | ORG | CON | ORG | CON | ORG | CON |
| Fruit fresh weight (g) | 27.3±2.14 | 34.2±2.64 | 21.4±1.07 | 29.9±4.06 | 29.1±3.12 | 29.5±1.60 |
| Dry matter (%) | 10.0±0.003 | 9.19±0.006 | 10.9±0.002 | 10.2±0.004 | 10.5±0.002 | 9.37±0.001 |
| Fruit weight loss (%) | 21.7±0.93 | 20.5±0.61 | 18.4±0.99 | 16.7±0.66 | 17.9±2.12 | 24.7±6.17 |
| Fruit firmness (N) | 7.83±0.51 | 7.92±0.15 | 5.44±0.39 | 6.06±0.28 | 6.47±0.99 | 6.23±0.51 |
| External L* (+60 to -60) | 39.2±0.99 | 40.3±0.21 | 38.6±0.30 | 39.2±0.11 | 35.3±1.37 | 36.3±0.83 |
| External C* (+60 to -60) | 44.3±1.32 | 44.1±0.21 | 43.4±0.37 | 42.6±0.84 | 38.8±2.00 | 36.8±0.07 |
| External h_ab_ (^o^) | 30.4±1.34 | 32.2±0.41 | 30.7±0.35 | 31.2±0.27 | 27.3±2.13 | 29.1±1.35 |
| Internal L* (+60 to -60) | 63.9±0.78 | 63.4±0.30 | 64.7±1.28 | 64.3±1.19 | 65.0±1.51 | 63.5±0.23 |
| Internal C* (+60 to -60) | 20.4±0.61 | 21.0±0.56 | 24.8±1.11 | 24.9±0.74 | 28.6±1.16 | 29.0±0.57 |
| Internal h_ab_ (^o^) | 59.2±1.17 | 58.9±1.11 | 55.8±2.16 | 54.9±1.57 | 54.6±0.33 | 54.8±0.93 |
| Soluble solids (^o^brix) | 9.45±0.28 | 7.44±0.22 | 9.31±0.83 | 9.63±0.69 | 9.03±0.23 | 8.77±0.44 |
| TA (mg citric acid g^-1^ FW) | 9.69±0.28 | 7.59±0.33 | 6.99±0.08 | 7.37±0.52 | 6.61±0.52 | 7.32±0.23 |
| Soluble solids/TA | 0.98±0.02 | 0.99±0.06 | 1.33±0.10 | 1.31±0.02 | 1.37±0.07 | 1.20±0.03 |
| pH | 3.54±0.02 | 3.59±0.01 | 3.80±0.08 | 3.85±0.01 | 3.90±0.003 | 3.89±0.02 |
| Reducing sugars (mg Glc g^-1^ FW) | 52.3±4.05 | 57.9±1.30 | 79.6±1.67 | 72.7±4.22 | 53.2±1.13 | 60.3±1.27 |
| Total sugars (mg Glc g^-1^ FW) | 54.7±0.80 | 63.8±0.27 | 94.4±6.53 | 90.4±4.55 | 63.5±1.26 | 68.6±0.03 |
| Total antioxidant activity  (mmol Trolox equiv/g FW)  Hydrophilic  Lipophilic | 12.1±0.62  9.60±0.45  2.50±0.17 | 11.1±1.10  8.88±0.84  2.25±0.28 | 13.3±0.67  10.9±0.44  2.38±0.27 | 11.0±0.94  9.17±0.75  1.84±0.23 | 9.88±1.35  8.16±0.90  1.73±0.45 | 11.1±1.31  9.44±1.07  1.62±0.25 |
| Total phenolics  (mg gallic acid equiv/g FW) | 1.22±0.03 | 1.14±0.04 | 1.33±0.04 | 1.24±0.03 | 1.27±0.02 | 1.17±0.01 |
| Total anthocyanins  (μg P-3-Glc^*^ equivalents/g FW) | 179±17.3 | 172±9.8 | 180±7.4 | 172±7.2 | 255±37.5 | 251±0.8 |
| Total ascorbic acid (mg/g FW)  Reduced AsA  Oxidized dehydro-AsA  Reduced AsA/total AsA | 0.58±0.02  0.46±0.04  0.11±0.01  0.78±0.04 | 0.48±0.06  0.38±0.07  0.11±0.03  0.78±0.08 | 0.82±0.05  0.60±0.02  0.22±0.04  0.73±0.04 | 0.69±0.06  0.50±0.01  0.19±0.06  0.73±0.06 | 0.64±0.04  0.52±0.06  0.11±0.04  0.82±0.04 | 0.78±0.01  0.60±0.04  0.18±0.03  0.78±0.05 |
| Ellagic acid (mg 100 g^-1^ FW)  Glycoside  Total | 1.36±0.64  37.9±8.21  39.2±8.77 | 7.66±7.02  38.3±6.04  46.0±5.05 | 1.52±0.03  95.9±11.2  97.4±11.2 | 1.34±0.24  81.3±35.7  82.7±35.8 | 1.59±0.25  42.2±26.2  43.8±26.4 | 1.03±0.04  113±13.1  114±13.1 |
| Quercetin (mg 100 g^-1^ FW)  Glycoside  Total | 2.88±0.15  4.97±0.84  7.85±0.75 | 2.81±0.20  7.70±0.72  10.5±0.55 | 2.85±0.07  10.0±0.56  12.9±0.51 | 2.99±0.28  6.29±2.16  9.28±2.15 | 2.46±0.18  9.79±0.22  12.3±0.40 | 2.71±0.04  8.38±1.11  11.1±1.07 |
| Kaempferol (mg 100 g^-1^ FW)  Glycoside  Total | 0.97±0.05  4.38±1.05  5.34±0.02 | 1.17±0.13  4.79±0.33  5.95±0.37 | 1.03±0.07  6.09±1.37  7.13±1.32 | 1.09±0.08  5.05±0.09  6.14±0.03 | 1.32±0.15  4.49±0.27  5.81±0.41 | 1.11±0.08  5.00±0.37  6.11±0.45 |
| Ploretin (mg 100 g^-1^ FW)  Glycoside (phloridzin)  Total | 2.31±0.05  1.00±0.31  3.32±0.32 | 3.00±0.33  2.19±1.15  5.19±0.90 | 2.55±0.07  3.53±0.97  6.09±0.90 | 2.42±0.10  3.12±0.75  5.54±0.66 | 2.17±0.11  3.75±1.90  5.92±1.79 | 2.37±0.13  3.97±0.29  6.33±0.43 |
| Naringenin (mg 100 g^-1^ FW)  S-enantomer  R-enantomer  Glycoside (naringin)  S-enantomer  R-enantomer  Total  S-enantomer  R-enantomer | 0.32±0.03  0.12±0.02  15.7±14.5  15.8±14.8  16.0±14.5  15.9±14.8 | 0.32±0.08  0.59±0.43  0.93±0.22  1.11±0.17  1.26±0.30  1.70±0.59 | 0.39±0.03  0.18±0.03  1.53±0.20  1.42±0.31  1.93±0.22  0.60±0.29 | 0.45±0.05  0.22±0.02  0.94±0.19  1.10±0.05  1.39±0.17  1.32±0.04 | 0.47±0.15  0.20±0.05  0.43±0.01  1.03±0.62  0.89±0.16  1.24±0.57 | 0.68±0.36  0.38±0.23  0.92±0.50  1.15±0.16  1.60±0.14  1.53±0.07 |
| Fruit nitrogen (% FW) | 1.01±0.06 | 1.14±0.08 | 1.02±0.02 | 1.15±0.01 | 1.29±0.00 | 1.27±0.09 |
| Fruit phosphorus (% FW) | 0.24±0.02 | 0.31±0.01 | 0.24±0.01 | 0.28±0.01 | 0.33±0.04 | 0.37±0.06 |
| Fruit potassium (% FW) | 1.55±0.05 | 1.70±0.08 | 1.39±0.04 | 1.52±0.04 | 1.60±0.09 | 1.73±0.05 |
| Fruit calcium (% FW) | 0.11±0.01 | 0.14±0.02 | 0.12±0.01 | 0.09±0.003 | 0.15±0.005 | 0.15±0.01 |
| Fruit magnesium (% FW) | 0.13±0.01 | 0.14±0.01 | 0.13±0.01 | 0.13±0.003 | 0.15±0.005 | 0.15±0.00 |
| Fruit boron (ppm) | 9.67±0.33 | 10.3±0.88 | 12.0±0.58 | 15.7±2.67 | 15.0±0.00 | 16.5±1.5 |
| Fruit zinc (ppm) | 8.00±1.73 | 8.67±1.20 | 9.33±0.88 | 9.67±1.20 | 14.5±0.50 | 11.5±0.50 |
| Leaf nitrogen (% FW) | 2.71±0.05 | 2.96±0.13 | 3.24±0.12 | 3.06±0.03 | 3.05±0.12 | 3.14±0.12 |
| Leaf phosphorus (% FW) | 0.35±0.03 | 0.49±0.02 | 0.39±0.01 | 0.54±0.09 | 0.51±0.03 | 0.65±0.03 |
| Leaf potassium (% FW) | 1.42±0.04 | 1.52±0.04 | 1.72±0.12 | 1.62±0.06 | 1.66±0.08 | 1.65±0.09 |
| Leaf calcium (% FW) | 0.81±0.08 | 1.26±0.03 | 1.04±0.16 | 1.08±0.09 | 0.97±0.17 | 0.90±0.02 |
| Leaf magnesium (% FW) | 0.30±0.01 | 0.38±0.02 | 0.35±0.03 | 0.39±0.03 | 0.31±0.02 | 0.37±0.00 |
| Leaf sulfur (% FW) | 0.18±0.003 | 0.20±0.01 | 0.27±0.06 | 0.31±0.05 | 0.28±0.07 | 0.28±0.01 |
| Leaf boron (ppm) | 26.0±2.52 | 33.7±2.33 | 41.0±4.73 | 58.7±12.8 | 38.5±2.50 | 31.5±0.50 |
| Leaf zinc (ppm) | 16.7±1.76 | 22.0±2.31 | 23.7±5.70 | 18.3±3.18 | 18.0±3.00 | 20.0±0.00 |
| Leaf manganese (ppm) | 201±43.8 | 342±255 | 124±47.2 | 241±117 | 186±43.0 | 566±402 |
| Leaf copper (ppm) | 4.00±0.58 | 4.67±0.33 | 5.00±0.58 | 3.67±0.33 | 4.00±0.00 | 4.00±1.00 |
| Leaf iron (ppm) | 261±61.7 | 227±33.2 | 233±36.2 | 213±12.4 | 190±48.5 | 246±44.5 |
| Overall acceptance^*^ | 6.37±0.18 | 5.97±0.23 | 6.43±0.37 | 6.32±0.06 | 6.38±0.22 | 6.31±0.43 |
| Flavor^*^ | 5.96±0.14 | 5.38±0.33 | 6.19±0.24 | 5.88±0.14 | 6.12±0.24 | 5.88±0.60 |
| Juiciness^*^ | 5.63±0.12 | 5.76±0.12 | 6.61±0.25 | 6.79±0.15 | 6.50±0.02 | 6.52±0.04 |
| Sweetness^*^ | 5.64±0.26 | 5.19±0.38 | 6.05±0.22 | 5.85±0.01 | 5.88±0.52 | 5.72±0.52 |
| Tartness^*^ | 4.61±0.42 | 4.71±0.33 | 4.80±0.20 | 4.88±0.32 | 4.72±0.20 | 4.94±0.14 |
| Appearance^*^ | 6.98±0.25 | 6.56±0.12 | 6.60±0.26 | 7.24±0.43 | 7.28±0.00 | 6.90±0.02 |

^*^Nine-point hedonic/intensity scale.

**Table S4D. Fruit, leaf, and sensory properties (mean ± standard error) for ‘Diamante’, ‘Lanai’, and ‘San Juan’ strawberry cultivars from organic (ORG) and conventional (CON) farms** in June 2005.

| Fruit, Leaf, and Sensory Properties (units) | ‘Diamante’ (n=3) | | ‘Lanai’ (n=3) | | ‘San Juan’ (n=2) | |
| --- | --- | --- | --- | --- | --- | --- |
|  | ORG | CON | ORG | CON | ORG | CON |
| Fruit fresh weight (g) | 24.6±2.47 | 25.3±1.25 | 23.2±0.70 | 25.7±1.62 | 28.1±3.00 | 33.5±1.10 |
| Dry matter (%) | 10.6±0.002 | 8.12±0.005 | 10.1±0.013 | 9.76±0.009 | 9.07±0.003 | 9.88±0.005 |
| Fruit weight loss (%) | 17.8±0.27 | 17.5±0.99 | 19.2±0.87 | 19.6±0.36 | 18.5±0.92 | 18.9±0.44 |
| Fruit firmness (N) | 8.68±0.55 | 6.79±0.43 | 4.74±0.27 | 4.86±0.21 | 5.67±0.18 | 5.20±0.20 |
| External L* (+60 to -60) | 36.8±0.48 | 39.4±0.28 | 35.3±0.37 | 35.9±0.66 | 33.9±0.96 | 34.6±0.91 |
| External C* (+60 to -60) | 46.8±1.31 | 46.9±0.13 | 43.6±0.80 | 43.1±0.10 | 38.8±0.51 | 39.2±0.57 |
| External h_ab_ (^o^) | 32.2±0.45 | 34.4±0.46 | 31.1±0.28 | 30.7±0.18 | 28.5±0.39 | 29.0±1.26 |
| Internal L* (+60 to -60) | 62.9±0.66 | 64.5±0.58 | 61.7±1.28 | 63.2±1.59 | 63.9±0.55 | 63.0±0.82 |
| Internal C* (+60 to -60) | 18.3±0.50 | 17.9±0.32 | 26.7±0.91 | 22.0±3.44 | 28.8±0.31 | 29.0±0.76 |
| Internal h_ab_ (^o^) | 59.0±1.48 | 60.4±1.35 | 53.2±2.20 | 58.2±5.89 | 52.7±0.57 | 53.9±0.36 |
| Soluble solids (^o^brix) | 9.45±0.28 | 7.44±0.22 | 9.31±0.83 | 9.63±0.69 | 9.03±0.23 | 8.77±0.44 |
| TA (mg citric acid g^-1^ FW) | 9.69±0.28 | 7.59±0.33 | 6.99±0.08 | 7.37±0.52 | 6.61±0.52 | 7.32±0.23 |
| Soluble solids/TA | 0.98±0.02 | 0.99±0.06 | 1.33±0.10 | 1.31±0.02 | 1.37±0.07 | 1.20±0.03 |
| pH | 3.54±0.02 | 3.59±0.01 | 3.80±0.08 | 3.85±0.01 | 3.90±0.003 | 3.89±0.02 |
| Reducing sugars (mg Glc g^-1^ FW) | 60.3±3.41 | 52.6±4.33 | 88.6±5.11 | 96.0±7.00 | 80.3±3.48 | 85.7±2.50 |
| Total sugars (mg Glc g^-1^ FW) | 64.9±1.65 | 55.0±4.78 | 97.6±10.6 | 96.7±5.27 | 65.5±2.79 | 73.3±7.89 |
| Total antioxidant activity  (mmol Trolox equiv/g FW)  Hydrophilic  Lipophilic | 13.2±0.47  10.2±0.24  3.00±0.39 | 10.7±0.51  8.39±0.54  2.29±0.09 | 13.9±2.00  11.3±1.28  2.62±0.72 | 12.8±1.89  10.6±1.49  2.25±0.40 | 11.1±1.86  9.33±1.35  1.75±0.51 | 11.3±1.36  9.51±1.22  1.79±0.14 |
| Total phenolics  (mg gallic acid equiv/g FW) | 1.39±0.03 | 1.10±0.04 | 1.28±0.07 | 1.17±0.07 | 1.03±0.07 | 1.03±0.01 |
| Total anthocyanins  (μg P-3-Glc^*^ equivalents/g FW) | 188±14.3 | 18±9.4 | 205±12.0 | 205±16.7 | 331±3.6 | 317±16.3 |
| Total ascorbic acid (mg/g FW)  Reduced AsA  Oxidized dehydro-AsA  Reduced AsA/total AsA | 0.54±0.05  0.40±0.01  0.14±0.04  0.75±0.05 | 0.44±0.02  0.32±0.02  0.12±0.01  0.72±0.03 | 0.71±0.04  0.52±0.03  0.19±0.01  0.73±0.01 | 0.63±0.09  0.47±0.06  0.15±0.03  0.76±0.03 | 0.64±0.14  0.44±0.12  0.21±0.01  0.67±0.05 | 0.61±0.01  0.44±0.002  0.17±0.01  0.72±0.01 |
| Ellagic acid (mg 100 g^-1^ FW)  Glycoside  Total | 1.95±0.82  25.2±11.8  27.2±12.6 | 2.41±0.43  9.66±1.63  12.1±1.24 | 3.89±0.41  82.6±12.8  86.5±12.8 | 2.78±0.97  101±26.7  104±27.7 | 3.87±0.82  117±9.95  120±9.13 | 2.46±0.41  68.5±53.2  71.0±52.8 |
| Quercetin (mg 100 g^-1^ FW)  Glycoside  Total | 2.81±0.22  7.78±1.41  10.6±1.19 | 3.05±0.07  8.95±0.76  12.0±0.73 | 3.33±0.17  3.72±0.28  7.05±1.67 | 2.98±0.14  6.94±1.75  9.92±1.67 | 3.09±0.05  5.52±0.19  8.61±0.24 | 3.07±0.05  5.90±2.10  8.97±2.16 |
| Kaempferol (mg 100 g^-1^ FW)  Glycoside  Total | 1.35±0.04  5.16±0.37  6.51±0.40 | 0.97±0.09  4.25±1.35  5.22±1.38 | 1.02±0.04  6.01±0.32  7.04±0.36 | 1.07±0.06  6.00±0.04  7.07±0.06 | 1.18±0.002  5.31±0.14  6.49±0.15 | 1.10±0.04  5.93±0.55  7.03±0.58 |
| Ploretin (mg 100 g^-1^ FW)  Glycoside (phloridzin)  Total | 2.45±0.22  3.43±1.28  5.88±1.22 | 2.44±0.04  1.76±0.62  4.20±0.58 | 2.70±0.05  3.09±0.75  5.79±0.75 | 2.54±0.03  2.99±0.55  5.53±0.52 | 2.68±0.18  3.31±0.65  6.00±0.47 | 2.59±0.05  3.08±0.06  5.68±0.01 |
| Naringenin (mg 100 g^-1^ FW)  S-enantomer  R-enantomer  Glycoside (naringin)  S-enantomer  R-enantomer  Total  S-enantomer  R-enantomer | 0.54±0.10  0.47±0.15  1.05±0.10  0.78±0.19  1.59±0.11  1.24±0.18 | 0.71±0.25  0.50±0.29  0.54±0.23  1.39±0.96  1.24±0.20  1.89±0.82 | 0.35±0.02  0.16±0.04  1.38±0.36  0.89±0.18  1.72±0.34  1.05±0.21 | 0.28±0.01  0.13±0.02  3.46±1.88  3.78±1.92  3.75±1.88  3.92±1.94 | 0.39±0.03  0.22±0.05  1.65±0.69  1.98±0.92  2.04±0.72  2.20±0.87 | 0.31±0.02  0.14±0.02  3.09±1.19  3.27±1.48  3.40±1.22  34.1±1.50 |
| Fruit nitrogen (% FW) | 0.98±0.03 | 1.09±0.05 | 1.05±0.07 | 1.11±0.08 | 1.42±0.02 | 1.34±0.04 |
| Fruit phosphorus (% FW) | 0.21±0.01 | 0.26±0.03 | 0.22±0.01 | 0.27±0.01 | 0.31±0.01 | 0.28±0.02 |
| Fruit potassium (% FW) | 1.38±0.11 | 1.62±0.09 | 1.35±0.06 | 1.59±0.06 | 1.70±0.07 | 1.56±0.02 |
| Fruit calcium (% FW) | 0.09±0.01 | 0.15±0.03 | 0.07±0.01 | 0.10±0.01 | 0.10±0.03 | 0.09±0.02 |
| Fruit magnesium (% FW) | 0.12±0.01 | 0.13±0.01 | 0.11±0.01 | 0.13±0.01 | 0.13±0.00 | 0.13±0.01 |
| Fruit boron (ppm) | 49.3±1.86 | 48.0±0.58 | 50.0±1.00 | 55.7±4.70 | 53.0±3.00 | 49.5±0.50 |
| Fruit zinc (ppm) | 8.67±0.67 | 9.67±0.88 | 9.33±0.67 | 11.0±2.08 | 13.0±4.00 | 11.5±0.50 |
| Leaf nitrogen (% FW) | 2.45±0.03 | 2.87±0.07 | 3.02±0.08 | 2.91±0.11 | 2.76±0.16 | 2.64±0.12 |
| Leaf phosphorus (% FW) | 0.25±0.01 | 0.36±0.02 | 0.38±0.01 | 0.43±0.03 | 0.37±0.04 | 0.36±0.02 |
| Leaf potassium (% FW) | 1.53±0.15 | 1.54±0.12 | 1.63±0.15 | 1.73±0.16 | 1.50±0.10 | 1.38±0.03 |
| Leaf calcium (% FW) | 1.26±0.11 | 2.06±0.18 | 1.28±0.13 | 1.37±0.10 | 1.48±0.36 | 1.11±0.06 |
| Leaf magnesium (% FW) | 0.36±0.07 | 0.47±0.01 | 0.40±0.02 | 0.40±0.05 | 0.36±0.04 | 0.41±0.05 |
| Leaf sulfur (% FW) | 0.24±0.02 | 0.19±0.01 | 0.21±0.01 | 0.20±0.01 | 0.19±0.02 | 0.19±0.02 |
| Leaf boron (ppm) | 44.7±1.86 | 41.0±1.53 | 58.7±7.31 | 53.3±10.9 | 47.0±5.00 | 37.0±2.00 |
| Leaf zinc (ppm) | 2.33±0.33 | 3.00±0.00 | 3.00±0.00 | 3.00±0.00 | 3.00±0.00 | 6.00±3.00 |
| Leaf manganese (ppm) | 402±282 | 340±93.5 | 101±26.4 | 93.0±8.54 | 110±4.00 | 176±90.0 |
| Leaf copper (ppm) | 2.33±0.33 | 1.67±0.67 | 4.33±0.33 | 4.33±0.33 | 2.50±0.50 | 2.50±0.50 |
| Leaf iron (ppm) | 241±16.7 | 207±29.2 | 209±27.2 | 277±33.7 | 240±19.5 | 196±10.5 |
| Overall acceptance^*^ | 6.17±0.15 | 5.45±0.32 | 6.16±0.39 | 6.31±0.13 | 6.08±0.08 | 6.56±0.00 |
| Flavor^*^ | 6.15±0.42 | 5.24±0.34 | 5.95±0.31 | 5.87±0.35 | 5.50±0.18 | 6.02±0.14 |
| Juiciness^*^ | 5.41±0.28 | 5.83±0.13 | 6.64±0.09 | 6.72±0.08 | 6.12±0.40 | 6.54±0.06 |
| Sweetness^*^ | 5.76±0.54 | 4.67±0.27 | 5.56±0.36 | 5.35±0.13 | 5.34±0.14 | 6.04±0.04 |
| Tartness^*^ | 5.03±0.33 | 5.23±0.22 | 4.63±0.35 | 4.84±0.26 | 4.60±0.04 | 4.36±0.28 |
| Appearance^*^ | 6.81±0.41 | 6.23±0.25 | 7.04±0.14 | 7.19±0.14 | 7.32±0.08 | 7.74±0.10 |

^*^Nine-point hedonic/intensity scale.

**Table S4E. Fruit and sensory properties (mean ± standard error) for ‘Diamante’, ‘Lanai’, and ‘San Juan’ strawberry cultivars from organic (ORG) and conventional (CON) farms** in September 2005.

| Fruit and Sensory Properties (units) | ‘Diamante’ (n=3) | | ‘Lanai’ (n=3) | | ‘San Juan’ (n=2) | |
| --- | --- | --- | --- | --- | --- | --- |
|  | ORG | CON | ORG | CON | ORG | CON |
| Fruit fresh weight (g) | 22.9±3.06 | 28.3±0.95 | 19.3±1.38 | 24.4±0.50 | 22.0±1.83 | 23.5±0.50 |
| Dry matter (%) | 10.4±0.002 | 7.93±0.005 | 9.75±0.001 | 9.31±0.007 | 8.98±0.003 | 9.40±0.006 |
| Fruit weight loss (%) | 24.9±0.89 | 31.4±1.32 | 23.6±2.17 | 27.4±4.14 | 22.7±3.75 | 24.3±1.08 |
| Fruit firmness (N) | 6.87±0.39 | 6.12±0.32 | 5.03±0.42 | 4.67±0.09 | 5.75±0.25 | 5.51±0.70 |
| External L* (+60 to -60) | 37.7±0.65 | 39.8±0.64 | 37.9±0.92 | 38.5±0.40 | 37.2±1.47 | 36.9±0.87 |
| External C* (+60 to -60) | 44.1±0.20 | 44.8±0.21 | 41.8±0.17 | 41.3±0.85 | 40.3±0.33 | 39.0±0.57 |
| External h_ab_ (^o^) | 30.7±0.32 | 34.8±0.22 | 32.0±0.77 | 32.4±0.70 | 31.9±0.98 | 29.8±1.15 |
| Internal L* (+60 to -60) | 55.8±2.29 | 59.4±1.37 | 60.2±0.98 | 59.3±1.74 | 62.1±0.40 | 59.6±0.12 |
| Internal C* (+60 to -60) | 23.8±0.40 | 20.5±0.94 | 23.1±0.73 | 24.3±0.84 | 27.2±0.18 | 28.3±0.71 |
| Internal h_ab_ (^o^) | 53.8±0.61 | 61.1±1.20 | 56.9±0.47 | 56.6±0.69 | 55.9±2.14 | 54.1±0.11 |
| Soluble solids (^o^brix) | 10.5±0.26 | 8.61±0.52 | 9.28±0.18 | 10.3±0.85 | 8.76±0.01 | 10.0±0.08 |
| TA (mg citric acid g^-1^ FW) | 8.41±0.60 | 6.67±0.29 | 7.57±0.30 | 7.79±0.30 | 7.68±0.75 | 8.82±0.04 |
| Soluble solids/TA | 1.27±0.07 | 1.34±0.07 | 1.23±0.04 | 1.32±0.09 | 1.15±0.11 | 1.14±0.004 |
| pH | 3.57±0.07 | 3.65±0.01 | 3.62±0.02 | 3.73±0.08 | 3.71±0.07 | 3.70±0.05 |
| Reducing sugars (mg Glc g^-1^ FW) | 68.8±3.33 | 57.2±5.05 | 75.5±4.20 | 75.6±9.22 | 64.7±1.28 | 59.1±3.10 |
| Total sugars (mg Glc g^-1^ FW) | 76.5±0.57 | 68.9±4.57 | 88.9±5.43 | 99.8±4.06 | 60.6±11.3 | 77.1±12.0 |
| Total antioxidant activity  (mmol Trolox equiv/g FW)  Hydrophilic  Lipophilic | 11.1±0.70  8.80±0.49  2.29±0.22 | 9.94±0.83  7.69±0.57  2.25±0.26 | 11.5±0.69  9.57±0.44  1.92±0.27 | 9.40±0.74  8.06±0.54  1.34±0.20 | 10.2±0.69  8.42±0.74  1.83±0.04 | 10.2±0.59  8.40±0.23  1.78±0.37 |
| Total phenolics  (mg gallic acid equiv/g FW) | 1.26±0.03 | 1.03±0.07 | 2.21±0.05 | 1.00±0.03 | 1.12±0.09 | 1.09±0.02 |
| Total anthocyanins  (μg P-3-Glc^*^ equivalents/g FW) | 186±15.8 | 132±5.3 | 217±14.3 | 194±17.7 | 289±15.6 | 281±9.2 |
| Total ascorbic acid (mg/g FW)  Reduced AsA  Oxidized dehydro-AsA  Reduced AsA/total AsA | 0.49±0.03  0.33±0.03  0.15±0.02  0.68±0.04 | 0.44±0.03  0.33±0.03  0.10±0.004  0.76±0.01 | 0.55±0.04  0.41±0.02  0.14±0.03  0.75±0.04 | 0.52±0.04  0.40±0.03  0.13±0.03  0.76±0.04 | 0.59±0.0002  0.46±0.01  0.13±0.01  0.77±0.01 | 0.57±0.01  0.45±0.01  0.12±0.02  0.79±0.03 |
| Ellagic acid (mg 100 g^-1^ FW)  Glycoside  Total | 8.21±1.94  36.1±5.96  44.3±4.32 | 10.5±2.13  9.97±4.32  20.5±3.09 | 7.33±2.30  37.7±15.1  45.0±16.1 | 10.8±2.11  42.4±8.40  53.2±7.12 | 7.10±1.97  42.4±12.4  49.5±14.4 | 8.28±1.03  30.9±19.4  39.1±18.3 |
| Quercetin (mg 100 g^-1^ FW)  Glycoside  Total | 2.38±0.04  6.45±0.44  8.82±0.48 | 2.43±0.08  4.79±0.90  7.22±0.94 | 2.59±0.27  4.74±0.96  7.32±0.69 | 2.45±0.05  4.78±0.38  7.23±0.38 | 2.38±0.01  4.98±1.03  7.36±1.02 | 2.25±0.18  8.66±3.26  10.9±3.45 |
| Kaempferol (mg 100 g^-1^ FW)  Glycoside  Total | 0.89±0.01  5.48±0.20  6.37±0.20 | 0.93±0.01  5.12±0.11  6.05±0.12 | 1.07±0.06  5.05±0.05  6.12±0.01 | 1.09±0.04  5.45±0.41  6.54±0.36 | 0.99±0.02  5.39±0.53  6.39±0.51 | 1.03±0.02  5.82±0.45  6.85±0.42 |
| Ploretin (mg 100 g^-1^ FW)  Glycoside (phloridzin)  Total | 2.25±0.05  1.39±0.08  3.64±0.09 | 2.31±0.06  0.96±0.24  3.28±0.29 | 2.25±0.13  0.96±0.43  3.22±0.37 | 2.24±0.04  1.40±0.09  3.64±0.05 | 2.28±0.03  0.56±0.08  2.84±0.11 | 2.17±0.02  1.47±0.11  3.63±0.09 |
| Naringenin (mg 100 g^-1^ FW)  S-enantomer  R-enantomer  Glycoside (naringin)  S-enantomer  R-enantomer  Total  S-enantomer  R-enantomer | 0.30±0.01  0.11±0.01  0.81±0.05  0.68±0.03  1.11±0.05  0.79±0.02 | 0.30±0.02  0.11±0.01  0.73±0.02  0.65±0.03  1.02±0.04  0.77±0.04 | 0.29±0.04  0.15±0.06  0.75±0.04  0.63±0.04  1.04±0.04  0.79±0.05 | 0.29±0.01  0.12±0.01  0.85±0.04  0.73±0.03  1.14±0.03  0.84±0.02 | 0.30±0.03  0.12±0.005  0.73±0.09  0.66±0.05  1.02±0.06  0.78±0.06 | 0.32±0.02  0.10±0.03  0.89±0.13  0.76±0.09  1.22±0.11  0.87±0.06 |
| Overall acceptance^*^ | 6.00±0.08 | 5.29±0.15 | 6.47±0.06 | 6.41±0.11 | 6.56±0.04 | 6.58±0.06 |
| Flavor^*^ | 5.83±0.44 | 5.05±0.21 | 6.09±0.07 | 6.01±0.11 | 6.16±0.08 | 6016±0.08 |
| Juiciness^*^ | 5.72±0.16 | 5.95±0.20 | 6.63±0.09 | 6.51±0.07 | 6.62±0.06 | 6.62±0.02 |
| Sweetness^*^ | 5.47±0.38 | 4.71±0.21 | 5.72±0.17 | 5.75±0.14 | 5.94±0.06 | 5.92±0.08 |
| Tartness^*^ | 5.19±0.24 | 5.00±0.10 | 5.28±0.08 | 5.24±0.04 | 5.28±0.08 | 5.32±0.08 |
| Appearance^*^ | 7.07±0.36 | 6.09±0.12 | 7.59±0.06 | 7.37±0.31 | 7.66±0.02 | 7.66±0.02 |

^*^Nine-point hedonic/intensity scale.
