# Supplementary material for: Fruit and Soil Quality of Organic and Conventional Strawberry Agroecosystems
Source: PLoS One. 2010 Sep 1;5(9):e12346. doi: 10.1371/journal.pone.0012346 (PMC2931688; doi:10.1371/journal.pone.0012346)
Supplement: Table S5 — Soil properties (mean ± standard error) at two depths (0–10 cm and 20–30 cm) from organic and conventional strawberry farms in June 2004 and June 2005. (0.09 MB DOC) [file pone.0012346.s005.doc]

**Table S5A. Soil properties (mean ± standard error) at two depths (0-10 cm and 20-30 cm) from organic and conventional strawberry farms in June 2004.**

| Soil Property | Organic  (0-10 cm) | Conventional  (0-10 cm) | Organic  (20-30 cm) | Conventional  (20-30 cm) |
| --- | --- | --- | --- | --- |
| Sand (g 100 g^-1^ soil) | 68.7±13.4 | 69.7±12.6 | 69.3±13.1 | 68.7±13.9 |
| Silt (g 100 g^-1^ soil) | 19.6±8.88 | 18.8±8.02 | 19.0±8.45 | 19.0±8.7 |
| Clay (g 100 g^-1^ soil) | 11.6±4.66 | 11.4±4.65 | 11.6±4.65 | 12.2±5.21 |
| Nitrate (mg kg^-1^ soil) | 30.0±17.1 | 28.4±13.1 | 26.6±7.78 | 13.4±7.24 |
| Ammonium (mg kg^-1^ soil) | 3.0±0.8 | 2.6±0.2 | 2.4±0.2 | 2.8±0.6 |
| Phosphorus (mg kg^-1^ soil) | 48.6±20.5 | 52.0±10.2 | 45.0±19.0 | 51.6±12.1 |
| Sulfur (mg kg^-1^ soil) | 124±47.8 | 188±88 | 118±67.8 | 73.6±34.8 |
| Boron (mg kg^-1^ soil) | 1.08±0.57 | 0.84±0.57 | 0.77±0.44 | 0.62±0.40 |
| Zinc (mg kg^-1^ soil) | 2.72±0.79 | 2.02±0.24 | 2.50±0.82 | 1.62±0.31 |
| Manganese (mg kg^-1^ soil) | 5.26±0.76 | 9.44±4.42 | 3.84±0.25 | 4.14±0.98 |
| Copper (mg kg^-1^ soil) | 1.32±0.67 | 1.02±0.37 | 1.48±0.69 | 1.00±0.38 |
| Iron (mg kg^-1^ soil) | 23.0±3.81 | 19.0±4.03 | 20.2±3.14 | 26.0±3.73 |
| Potassium (cmol kg^-1^ soil) | 0.4±0.1 | 0.4±0.1 | 0.5±0.2 | 0.4±0.1 |
| Calcium (cmol kg^-1^ soil) | 8.3±3.8 | 7.2±2.6 | 8.2±4.2 | 6.7±2.8 |
| Magnesium (cmol kg^-1^ soil) | 3.8±2.4 | 4.2±3.0 | 3.4±2.3 | 4.0±3.0 |
| Sodium (cmol kg^-1^ soil) | 0.5±0.1 | 0.3±0.1 | 0.3±0.1 | 0.2±0.1 |
| Total bases (cmol (+) kg^-1^) | 13.0±6.4 | 12.1±5.8 | 12.3±6.8 | 11.4±5.9 |
| pH | 7.22±0.14 | 7.20±0.17 | 7.02±0.16 | 7.12±0.22 |
| Buffer capacity pH | 7.52±0.02 | 7.54±0.04 | 7.52±0.02 | 7.50±0.03 |
| EC (mmhos cm^-1^) | 2.33±0.64 | 2.69±0.97 | 2.11±0.79 | 1.56±0.44 |
| Total carbon (g kg^-1^ soil) | 8.24±0.28 | 7.37±0.20 | 7.69±0.32 | 6.65±0.21 |
| Total nitrogen (g kg^-1^ soil) | 0.650±0.027 | 0.544±0.018 | 0.603±0.030 | 0.477±0.020 |
| Readily mineralizable carbon (μg MinC g^-1^ soil) | 19.3±1.04 | 16.7±2.43 | 14.9±2.30 | 11.3±1.80 |
| Dehydrogenase (μg TPF g^-1^ soil) | 1.15±0.19 | 0.66±0.11 | 0.68±0.11 | 0.41±0.08 |
| Acid phosphatase (μg p-nitrophenol g^-1^ soil) | 127.2±35.5 | 59.0±11.3 | 104.7±37.4 | 53.1±9.1 |
| Alkaline phosphatase (μg p-nitrophenol g^-1^ soil) | 104.2±20.7 | 51.6±18.0 | 84.4±17.0 | 47.0±13.0 |
| Protease native (μg amino acid-N g^-1^ soil h^-1^) | 1.81±0.26 | 2.31±0.18 | 2.08±0.29 | 1.25±0.35 |
| Protease potential (μg amino acid-N g^-1^ soil h^-1^) | 3.09±0.48 | 3.38±0.37 | 3.21±0.25 | 2.78±0.31 |

**Table S5B. Soil properties (mean ± standard error) at two depths (0-10 cm and 20-30 cm) from organic and conventional strawberry farms in June 2005.**

| Soil Property | Organic  (0-10 cm) | Conventional  (0-10 cm) | Organic  (20-30 cm) | Conventional  (20-30 cm) |
| --- | --- | --- | --- | --- |
| Sand (g 100 g^-1^ soil) | 51.8±9.72 | 51.3±10.5 | 52.7±10.1 | 50.9±11.1 |
| Silt (g 100 g^-1^ soil) | 33.9±4.96 | 34.0±6.90 | 32.6±5.55 | 35.7±6.87 |
| Clay (g 100 g^-1^ soil) | 14.3±4.98 | 14.7±3.85 | 14.8±5.03 | 13.5±4.29 |
| Nitrate (mg kg^-1^ soil) | 63.6±15.5 | 34.9±9.22 | 22.4±4.45 | 32.4±9.93 |
| Ammonium (mg kg^-1^ soil) | 2.5±0.3 | 3.2±0.4 | 2.6±0.3 | 2.7±0.3 |
| Phosphorus (mg kg^-1^ soil) | 73.3±17.9 | 77.0±9.79 | 75.3±18.2 | 92.6±12.4 |
| Sulfur (mg kg^-1^ soil) | 144±41.4 | 49.9±14.8 | 120±51.8 | 37.9±10.4 |
| Boron (mg kg^-1^ soil) | 0.68±0.15 | 0.64±0.24 | 0.65±0.18 | 0.88±0.44 |
| Zinc (mg kg^-1^ soil) | 3.04±0.39 | 1.91±0.14 | 2.34±0.39 | 2.00±0.34 |
| Manganese (mg kg^-1^ soil) | 3.79±0.61 | 5.84±1.89 | 2.43±0.49 | 3.21±0.47 |
| Copper (mg kg^-1^ soil) | 1.41±0.32 | 1.31±0.33 | 1.31±0.31 | 1.45±0.41 |
| Iron (mg kg^-1^ soil) | 34.3±5.59 | 34.6±7.15 | 32.5±4.46 | 36.9±8.42 |
| Potassium (cmol kg^-1^ soil) | 0.8±0.1 | 0.6±0.1 | 0.8±0.1 | 0.6±0.2 |
| Calcium (cmol kg^-1^ soil) | 13.1±2.9 | 12.2±2.9 | 12.4±3.1 | 12.4±3.0 |
| Magnesium (cmol kg^-1^ soil) | 4.4±1.2 | 4.3±1.3 | 4.4±1.3 | 4.4±1.3 |
| Sodium (cmol kg^-1^ soil) | 0.4±0.1 | 0.3±0.04 | 0.3±0.04 | 0.30±0.04 |
| Total bases (cmol (+) kg^-1^) | 18.7±4.2 | 17.3±4.2 | 17.9±4.6 | 17.7±4.4 |
| pH | 6.89±0.14 | 6.99±0.26 | 7.30±0.12 | 7.05±0.26 |
| Buffer capacity pH | 7.50±0.03 | 7.49±0.04 | 7.54±0.03 | 7.54±0.04 |
| EC (mmhos cm^-1^) | 3.09±0.38 | 1.68±0.16 | 2.14±0.40 | 1.44±0.25 |
| Total carbon (g kg^-1^ soil) | 11.83±0.17 | 9.13±0.16 | 11.16±0.19 | 8.78±0.16 |
| Total nitrogen (g kg^-1^ soil) | 1.081±0.012 | 0.791±0.013 | 0.963±0.016 | 0.773±0.014 |
| Readily mineralizable carbon (μg MinC g^-1^ soil) | 16.1±1.50 | 11.4±0.76 | 14.9±2.37 | 11.2±1.74 |
| Microbial biomass (μg MicC g^-1^ soil) | 249±22.5 | 96±6.8 | 211±20.5 | 101±12.1 |
| MicC (% of total carbon) | 2.21±0.13 | 1.33±0.26 | 2.16±0.31 | 1.54±0.37 |
| MicC MinC^-1^ | 16.0±1.8 | 8.6±0.6 | 16.8±3.1 | 9.3±0.5 |
| Basal respiration (μg CO_2_-C g^-1^ soil h^-1^) | 0.472±0.055 | 0.354±0.032 | 0.731±0.186 | 0.348±0.111 |
| Dehydrogenase (μg TPF g^-1^ soil) | 1.61±0.31 | 0.64±0.05 | 1.10±0.20 | 0.63±0.05 |
| Acid phosphatase (μg p-nitrophenol g^-1^ soil) | 115.8±9.8 | 57.4±6.4 | --- | --- |
| Alkaline phosphatase (μg p-nitrophenol g^-1^ soil) | 140.3±15.7 | 59.6±9.0 | --- | --- |
| qCO_2_ (ug CO_2_-C h^-1^ ug^-1^ MicC)^§^ | 0.0019±0.00018 | 0.0037±0.00033 | 0.0035±0.00073 | 0.0034±0.00067 |
| Protease native (μg amino acid-N g^-1^ soil h^-1^) | 3.02±0.36 | 3.30±0.55 | --- | --- |
| Protease potential (μg amino acid-N g^-1^ soil h^-1^) | 5.02±0.95 | 3.61±0.48 | --- | --- |
| Mycorrhizae total colonized root length (mm)^§^ | 122±11 | 104±10 | --- | --- |
